# Importance of structure-based studies for the design of a novel HIV-1 inhibitor peptide

**DOI:** 10.1038/s41598-020-71404-0

**Published:** 2020-09-02

**Authors:** María J. Gomara, Yolanda Perez, Patricia Gomez-Gutierrez, Carolina Herrera, Paul Ziprin, Javier P. Martinez, Andreas Meyerhans, Juan J. Perez, Isabel Haro

**Affiliations:** 1 Unit of Synthesis and Biomedical Applications of Peptides, IQAC-CSIC, Jordi Girona, 18-26, 08034 Barcelona, Spain; 2 Nuclear Magnetic Resonance Facility, IQAC-CSIC, Jordi Girona, 18-26, 08034 Barcelona, Spain; 3 Department of Chemical Engineering (ETSEIB), Universitat Politecnica de Catalunya, Barcelona, Spain; 4 Department of Medicine, Imperial College London, London, UK; 5 Department of Surgery and Cancer, St. Mary’s Hospital, Imperial College London, London, UK; 6 Infection Biology Laboratory, Department of Experimental and Health Sciences, Universitat Pompeu Fabra, Barcelona, Spain; 7 ICREA, Pg. Lluís Companys 23, 08010 Barcelona, Spain

**Keywords:** Biophysics, Chemical biology, Diseases

## Abstract

Based on the structure of an HIV-1 entry inhibitor peptide two stapled- and a retro-enantio peptides have been designed to provide novel prevention interventions against HIV transmission. The three peptides show greater inhibitory potencies in cellular and mucosal tissue pre-clinical models than the parent sequence and the retro-enantio shows a strengthened proteolytic stability. Since HIV-1 fusion inhibitor peptides need to be embedded in the membrane to properly interact with their viral target, the structural features were determined by NMR spectroscopy in micelles and solved by using restrained molecular dynamics calculations. Both parent and retro-enantio peptides demonstrate a topology compatible with a shared helix–turn–helix conformation and assemble similarly in the membrane maintaining the active conformation needed for its interaction with the viral target site. This study represents a straightforward approach to design new targeted peptides as HIV-1 fusion inhibitors and lead us to define a retro-enantio peptide as a good candidate for pre-exposure prophylaxis against HIV-1.

## Introduction

The therapeutic utility of synthetic peptides as Human Immunodeficiency Virus (HIV) fusion inhibitors is largely limited due to their natural proteogenic structure, which is easily recognized and degraded by proteolytic enzymes. This limitation has hampered the clinical use of T20, a 36-mer peptide that binds to the HIV-1 gp41, and requires high dosing and repeated injections due to its lack of oral bioavailability^1,2^.

Among the different strategies studied to improve the pharmacokinetic behaviour of peptides composed of natural amino acids, the synthetic d-peptides have demonstrated a higher enzymatic stability, an increased serum half-life and the possibility to be absorbed systemically when taken orally^3,4^. d-amino acids decrease the substrate recognition and binding affinity of proteolytic enzymes and confer enzymatic stability to d-peptides^5–7^. In addition, it has been recently reported that d-peptides derived from a parent sequence not related to any existing protein silence the immune system and avoid specific humoral responses. Thus retro-D peptides, which display a similar topological arrangement as their parent peptides, offer the advantage of overcoming immunological problems when they are used as therapeutic agents^8^. Despite these advantages, a limited number of studies have addressed the therapeutic usefulness of d-peptide structures as HIV-1 fusion inhibitors^9^. However, it is worth mentioning^10,11^ the cyclic and trimeric d-peptide inhibitors that target the gp41 coiled-coil pocket as well as their subsequent modifications to increase the pharmacokinetics profiles and anti-viral activity^12,13^.

Alternatively to the use of non-natural amino acids, the introduction of conformational restrictions in linear peptide sequences has been widely used as an strategy to overcome peptide flexibility improving their biological potency^14^. Particularly, it has been described the stapling technique which consists on the covalent crosslinking of residues found on the same side of an α-helix as an strategy to locked short linear peptides into their bioactive α-helix secondary structure. Stapled peptides have shown an increased affinity to their target, a higher resistance to proteolytic digestion and in the end, an improved pharmacologic performance^15–17^.

On the basis of the structural knowledge of a previously defined peptide (L-E1P47) as an HIV-1 fusion inhibitor, this work describes the design and synthesis of new optimized peptide analogs by using either non-natural d-amino acids or the stapling technique. We synthesized a peptide composed of d-amino acids assembled in the reverse order to that of the parent L-sequence, in order to maintain the side-chain topology of the l-peptide^18–20^. Additionally, to reinforce the helicity on N- or C-terminus of L-E1P47 peptide, stapled peptides were synthesized by lactamisation between the amine- and carboxy-side chains of Lys and Asp or Glu amino acids. The conformational features of the synthesized analogues were comparatively studied by Circular Dichroism (CD) and Nuclear Magnetic Resonance (NMR) spectroscopy on DPC micelles. To envisage the in vitro half-life of the peptides, their proteolytic stability in human serum was analysed. In addition, their anti-viral activity was tested in cells and in an ex vivo human mucosal tissue models to gain more knowledge about possible therapeutic application of the synthesized peptides. To get further into the structural features of the most active and proteases-resistant peptide, NMR and computational modelling using the experimental NOE data as distance restrains were addressed. Finally, taking into account that fusion inhibitor peptides specifically interfering with the N-terminal region of gp41^21^ need to be embedded into the membrane in order to properly interact with their viral target^22^, we also studied the peptide assembly on the membrane as well as the recognition of its viral target on a membrane mimetic environment.

## Results and discussion

### Optimization of the lead peptide E1P47 by designing a retro-enantio and stapled analogues

**Figure 1:**
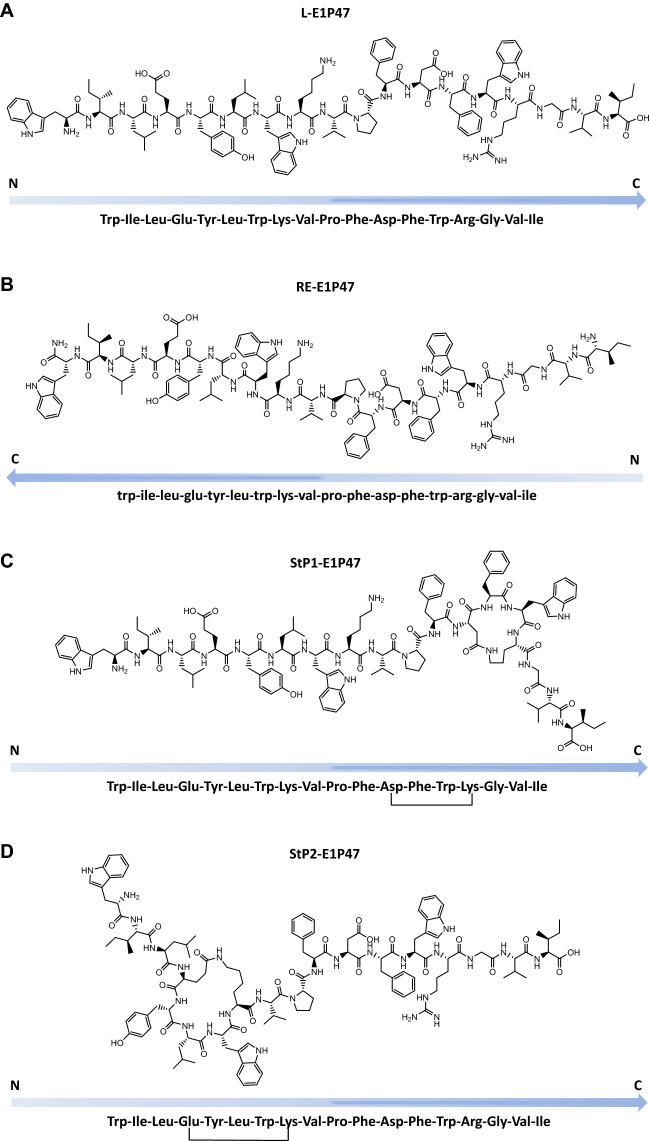
Primary structure of L-E1P47 (**A**), RE-E1P47 (**B**) and stapled peptides StP1-E1P47 (**C**) and StP2-E1P47 (**D**). Software ChemDraw Professional 16 (https://sitelicense.cambridgesoft.com/sitelicense.cfm?sid=55) was used.

Previous work of our group defined a new peptide lead, namely E1P47, as an entry inhibitor with a broad spectrum activity against HIV-1^23^. Particularly, E1P47 derives from the region 139–156 of the E1 protein of the Human Pegivirus which can be considered as commensal of humans since infections provide a beneficial effect on survival in HIV-1 positive subjects^24^. Several experimental data demonstrated that E1P47 can be considered as an inhibitor of HIV-1 Env fusion; (1) the peptide inhibited HIV-1 Env mediated cell fusion^23^; (2) the peptide was not able to inhibit an amphotropic vesicular stomatitis virus (VSV) Env pseudotyped on an HIV-1 core^23^ (3) peptide–peptide titrations and diffusion NMR spectroscopy demonstrated the specific interaction of E1P47 peptide with its viral target site, the HIV-1 fusion peptide^25^. In addition, reported structural work in DPC micelles demonstrated the importance of specific structural features for the anti-HIV-1 broad-spectrum potency of E1P47^25^. Two α-helices in both N- and C-terminal regions of the inhibitor peptide separated by a hinge region featured by a Pro residue were identified as structural elements required for maintaining the antiviral activity. Based on this structural background, in this work we propose the design and synthesis of new optimized analogs for increasing functionality in terms of efficiency and stability (Fig. 1). Since the arrangement of d-amino acids in a reverse sequence to the l-parent peptide can lead to a conformation that achieves a good mimicry with the l-peptide^8^, the retro-enantio version of the E1P47 (RE-E1P4) peptide was synthesized (Fig. 1B). Alternatively, to reinforce the α-helical conformation either on N- or C-terminus of L-E1P47, we designed two stapled peptides based on the previous structural study of the parent peptide^25^ which clearly demonstrated the existence of salt bridges between the Glu-4 and Lys-8 residues as well as between the Asp-12 and Arg-15 residues that favored the helical structures in the N and C-terminal segments. Thus, a stapled peptide was synthesized with a lactam bridge between Asp-12 and a Lys-15 that substituted the original Arg residue in order to stabilize the C-terminal α-helix (StP1-E1P47) and a lactam bond was formed between the residues Glu-4 and Lys-8 to obtain a cyclized version of the parent peptide, namely StP2-E1P47 (Fig. 1C,D).

**Figure 2:**
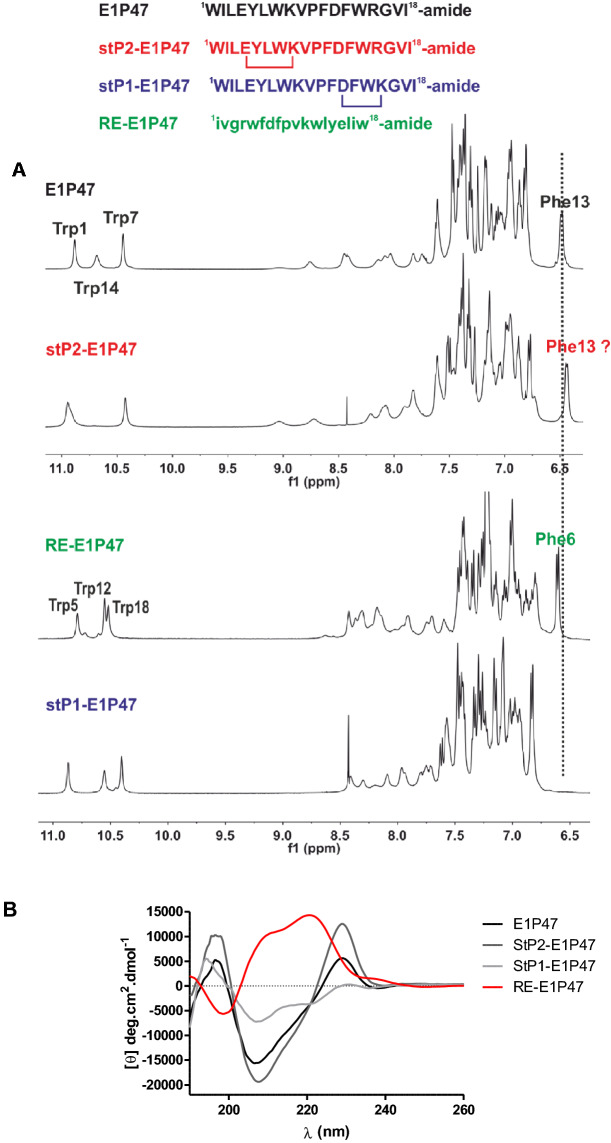
(**A**) Comparison of aromatic/amide region of ^1^H spectra of E1P47 and its stapled version StP2-E1P47 (with a bridge in N-terminal side) (top) and retro-enantio E1P47 and stapled peptide StP1-E1P47 (with a bridge in C-terminal side). Spectra were acquired in 100:1 DPC-d_38_:peptide, in 15 mM HEPES-d_11_, 90% H_2_O/10% D_2_O, pH 6.2, at 308 K. MNova 12.0.0 software for Windows (Mestrelab Research S.L., Santiago de Compostela, Spain) was used (**B**) Circular dichroism spectra of the peptides in DPC micelles. Peptide concentration was 70 µM and the peptide:DPC ratio was 1:100. Software GraphPad Prism 5.0 (https://graphpad-prism.software.informer.com/5.0/) was used.

First, we comparatively studied the conformational features of the synthesized analogues. Structural qualitative information for the stapled peptides was obtained by 1D ^1^H NMR (Fig. 2A) on DPC micelles. The ^1^H NMR spectrum of StP2-E1P47 resembles that of E1P47, with similar dispersion of NH amide (between 7.5 and 9.1) and indole protons (two NH indole Trp side chains are overlapped in proton spectrum, Fig. 2A), whereas the spectrum of StP1-E1P47 shows lower dispersion of amide protons.

Our previous studies showed that the E1P47 structure is stabilized by a quadrupole–quadrupole interaction between the two aromatic side chains of Phe-13 and Trp-14, giving a characteristic upfield shift of Phe-13 aromatic protons. The presence of this characteristic aromatic upfield shift was also observed in StP2-E1P47 and the RE-E1P47 but not in the StP1-E1P47 proton spectra. This lack of a shielding effect in the stapled peptide could be related to the disappearance of the electronic effect on the chemical shift due to the change of the relative orientation between Phe-13/Trp-14 aromatic side chains, due to the stiffness of the peptide backbone.

In addition, the secondary structure of stapled peptides and RE-E1P47 was studied by circular dichroism (CD) on DPC micelles and compared to that of the l-parent peptide E1P47. As described previously^25^ the CD spectrum of E1P47 peptide in DPC micelles showed bands located between 225 and 230 nm that could be attributed to interaction of the aromatic rings of Trp with the peptide backbone and stacking of aromatic rings^26^. In agreement to the NMR study, the band located at 229 nm could be related to the quadrupole–quadrupole interaction between the two aromatic chains of Phe-13 and Trp-14. As described by Woody et al.^27^ this band can be either positive or negative depending on the orientation of the Trp aromatic rings relative to the peptide backbone.

The α_R_ conformation is predicted to give rise to moderately strong positive bands located near 225 nm. Thus, the CD spectrum of E1P47 exhibited a positive CD band at 229 nm that can be attributed to the contribution of the Trp indole rings when these are located close to a α_R_ region. Moreover, the negative band at 208 nm reinforces the hypothesis of an α-helix as the main secondary structural component in the peptide. Similarly, the CD spectrum of StP2-E1P47 exhibits a stronger positive band at 229 nm and a more negative band at 208 nm (Fig. 2B). Accordingly, stabilization of the α-helix at the N-terminus did not seem to change the stacking of the two aromatic chains of Phe-13 and Trp-14, leading to a similar conformational profile as E1P47. In contrast, the CD spectrum of StP1-E1P47 while conserving the negative band at 208 nm, it did not exhibit a positive band at 229 nm, suggesting that although the peptide retained its helical conformation, the relative orientation between Phe-13/Trp-14 aromatic side chains was different to that observed in E1P47 and StP2-E1P47. Thus, the tethering of Asp-12 and Arg-15 at the C-terminus represented a more substantial change for the aromatic side chains orientation in the micellar environment. These CD results were totally consistent with the above chemical shift differences obtained by RMN. In addition, the CD spectrum of StP1-E1P47 exhibited characteristics associated with a 3_10_-helix conformation with a ratio between 222 and 207 nm bands of approximately 0.4^28,29^. The tethering between residues *i* (Asp12) and *i* + 3 (Lys15) on StP1-E1P47 could favor the formation of an hydrogen bond between *i* carbonyl and *i* + 3 amide NH, compatible with the observation of a 3_10_-helix on CD spectrum, which is slightly tighter than an α-helix. Also, this could affect the relative orientation of Phe13 and Trp14 aromatic side chains. Lastly, the CD spectrum of the RE-E1P47 showed characteristics of α-helical conformation but in its specular image (left-handed α-helix) since it corresponds to a d-peptide (Fig. 2B).

**Figure 3:**
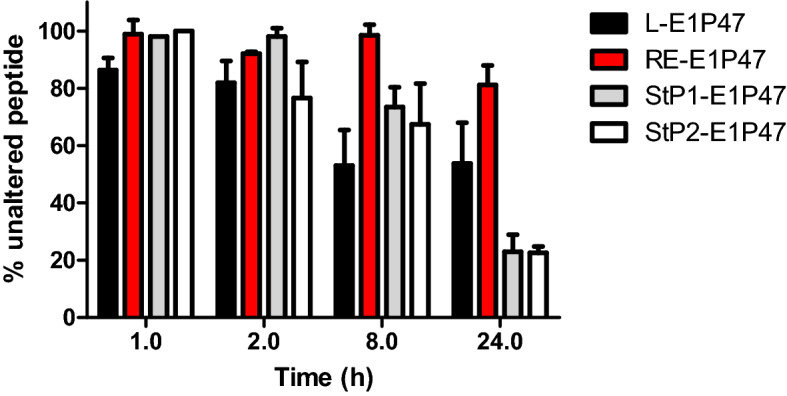
Representation of the percentage of peptide that remains in solution with respect to the initial one at different times. The percentage is calculated by dividing the area (HPLC analysis) at time “t” by the initial area of the peptide in the test conditions. Bar errors represents SEM of three replicates. Software GraphPad Prism 5.0 (https://graphpad-prism.software.informer.com/5.0/) was used.

In addition to the analysis of conformational features of the peptides, their susceptibility to human plasma proteases was also comparatively analysed. After incubation of each peptide with human plasma at 37 °C, RE-E1P47 remained practically unaltered for almost 24 h, meanwhile the L-parent peptide was about 50% degraded at 8 h. The cyclic nature of stapled peptides was expected to hamper the action of proteases^30^ and in fact, in our hands they presented higher stability compared to the parent peptide but lower than that of the retro-enantio peptide and after 24 h of incubation they exhibited even more dramatic protease lability (Fig. 3). Although the stapled peptides showed higher stability at the initial hours of incubation, after 24 h they were degraded by around 80%.

To better understand the metabolically vulnerable points of the peptides, we carried out an ultra-performance liquid chromatography-mass spectrometry (UPLC-MS) analysis of the major degradation products at different times of serum incubation. According to the predicted cleavage sites by the PROSPER bioinformatics program^31^ (Supplementary Table S1) the main metabolites that were identified by mass spectrometry suffered from enzymatic hydrolysis of Glu-4 (cathepsin K), Gly-16 (matrix metallopeptidase), Trp-1 and Phe-11 (chymotrypsin A) and Trp-7 (cathepsin G). The degradation product of cathepsin G was observed in StP1-E1P47 but not in StP2-E1P47. This differential behavior could be attributed to the presence of the intramolecular cycle established between positions Glu-4 and Lys-8 in StP2-E1P47, which could confer inaccessibility to degradation by cathepsin G of Trp-7. Similarly, the product of degradation of Phe-11 by chymotrypsin A was only observed in StP2-E1P47, since in StP1-E1P47 the cleavage site is next to the cycle established between Asp-12 and Lys-15. It should be noted that all of these metabolites were observed for the L-E1P47; however, its retro-enantio analogue did not suffer most of these proteolytic degradation. The introduction of non-natural amino acids would hamper the action of proteases clearly contributing to RE-E1P47 enhanced stability.

### Peptides candidates inhibit HIV-1 infection in pre-clinical cellular and mucosal tissue models

Epidemiological and genetic studies have shown that > 95% of sexually transmitted infections world-wide are due to R5-tropic viruses^32–34^. Hence, taking into account the predominant transmission of R5-tropic isolates compared with X4-viruses during sexual intercourse and our previous studies evaluating the potency of prototype fusion inhibitor T20^35^, we assessed the potency of the fusion inhibitor candidates against an R5-tropic isolate commonly used in pre-clinical studies, HIV-1_BaL_. The wild type (E1P47) and derivative peptides (RE-E1P47, StP1- E1P47 and StP2- E1P47) were first tested in TZM-bl cells. The three derivative peptides strongly inhibited HIV-1_Bal_ infection (Table 1, Supplementary Figure S1). RE-E1P47 showed about 19-fold improvement over L-E1P47 antiviral activity while StP1-E1P47 and StP2-E1P47 showed about seven and fivefold improvement, respectively. Thus, the RE-E1P47 was the most active analogue with an average IC_50_ value in the range of nanomolar concentration.

**Table 1:** Activity (IC_50_ values in µM) of E1P47-derived peptides against HIV-1_BaL_.

Model and peptide exposure	E1P47	RE-E1P47	StP1-E1P47	StP2-E1P47
TZM-bl cells sustained	0.76 ± 0.08	0.04 ± 3 × 10^–5^	0.11 ± 0.01	0.16 ± 0.01
Colorectal explants pulse	38.5 ± 17.2	28.7 ± 23.2	33.0 ± 27.4	32.0 ± 15.5
Colorectal explants sustained	8.0 ± 4.6	3.7 ± 1.7	2.8 ± 0.5	2.9 ± 0.4

The IC_50_ values shown are the means ± SEM derived from three independent experiments for each condition performed in triplicate.

The inhibitory activity of the four peptides was then assessed in a mucosal model based on ex vivo HIV-1 challenge of colorectal tissue explants^36,37^. Mucosal tissue explant models are becoming an important tool for pre-clinical screening of pre-exposure prophylaxis (PrEP) candidates and are increasingly used in early clinical trials^38–41^. These models assess the anti-viral potency of drugs candidates at the mucosal portal of HIV-1 transmission. Colorectal explants were treated with peptides before and during viral exposure (3 h) as a “pulse” condition to mimic drug dosing immediately prior to intercourse and during exposure; or throughout the culture as a “sustained” exposure to mimic the activity of the peptides when delivered from a sustained release formulation, such as an intravaginal ring. A dose–response curve was observed for all peptides in colorectal tissue explants against HIV-1_BaL_ with both treatment conditions, pulse and sustained increased the inhibitory potency of the four peptides with a decrease of IC_50_ values (Table 1).

RE-E1P47 tended to reach higher levels of inhibition in both dosing conditions at the highest concentration tested; however, no significant differences were observed in the inhibitory potency of the four peptides in this tissue model (Supplementary Figure S1). Sustained exposure to peptides increased the inhibitory potency of the four peptides with a decrease of IC_50_ values (Table 1). One limitation of our study is the absence of peptide controls. Due to the SARS-CoV-2 pandemic new experiments are a significant undertaking that requires access to laboratories that are currently closed. This includes containment level 3 laboratories (CL3) for live HIV infection experiments of cells and tissue samples.

The restriction of the conformational flexibility on N- or C-terminal end of L-E1P47 by the tethering of the residues involved in the formation of salt bridges stabilizing α-helical conformations led to an increase of the anti-viral potency of the parent peptide. Moreover, RE-E1P47 that showed a characteristic left-handed α-helix by circular dichroism was the most active peptide in cellular assays and demonstrated a tendency to reach higher levels of viral inhibition in the tissue model. In addition to the peptide conformation, peptide susceptibility to proteases also determined the inhibitory potency of the analogues. Unlike, L-and stapled E1P47 peptides, the RE-E1P47 was highly resistant to human proteases. Thus, both conformation features and proteolytic stability mostly determines the functionality of the RE-E1P47.

### RE-E1P47 assembles into the membrane maintaining the active conformation needed for its interaction with the HIV-1 target

Once selected RE-E1P47 as the most active and proteases-resistant peptide, a further structural characterization of the retro-enantio peptide as well as its assembly into the membrane and its subsequent ability to recognize the HIV-1 target were studied.

Structural studies of the RE-E1P47 in DPC micelles were carried out by NMR spectroscopy and computational modelling using the experimental NOE data as distance restrains. Proton chemical shift assignments of RE-E1P47 in DPC micelles (2 mM, at 308 K in aqueous solution with DPC-d_38_ at a detergent/protein ratio of ~ 100) were obtained using standard methods of peptide NMR spectroscopy as 2D NMR ^1^H–^1^H TOCSY, NOESY and ROESY experiments (Supplementary Table S2). The summary of the sequential and medium-range NOEs for the RE-E1P47 peptide in a solution of H_2_O/D_2_O with DPC-d_38_ micelles is shown in Supplementary Figure S2.

NMR experiments revealed several sets of conformations in slow exchange in chemical shift timescale for the retro-enantio peptide; however the broad resonances due to the presence of and peptide interaction with DPC micelles prevented the complete assignment of the NOE crosspeaks from minor conformations due to resonance overlapping.

**Figure 4:**
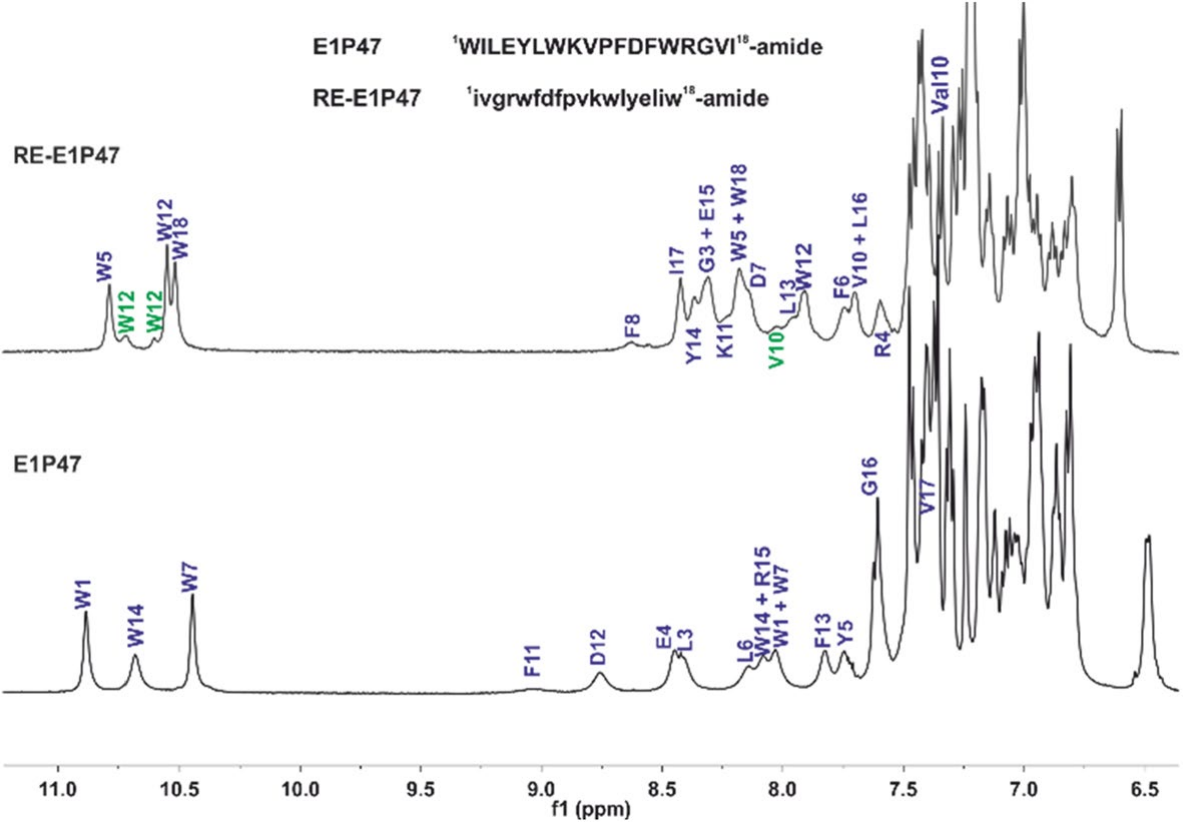
Assigned amide region of ^1^H spectra of E1P47 (bottom, from Ref.^25^) and its retro-enantio version (top, this work) in 100:1 DPC-d_38_:peptide (15 mM HEPES-d_11_, 90% H_2_O/10% D_2_O, pH* 6.2, at 308 K). Peaks labeled in blue are from the major peptide conformation and peaks labeled in green are for minor peptide conformations. MNova 12.0.0 software for Windows (Mestrelab Research S.L., Santiago de Compostela, Spain) was used.

For some amino acids, these resonances were clearly resolved, like the three components of Trp-12 (Fig. 4) or the tripling of Val-10 methyl protons resonances, whereas methyl proton’s from other residues showed no additional set of signals (Supplementary Figure S3). The chemical shifts of the additional signals could be reproduced in several sample preparations and were not concentration-dependent (^1^H NMR spectra of 2.0 and 0.2 mM RE-E1P47 in DPC-d_38_ micelles were identical, data not shown). One explanation for the observation of several sets of spin systems may be cis–trans isomerization of RE-E1P47 central proline. One of the new characteristics of RE-E1P47 compared to the parent peptide is the location of a phenylalanine (Phe-8) just before the proline residue. It is known that aromatic and prolines residues can interact locally and stabilize cis-prolyl-amide bonds, in particular aromatic–proline sequences, via both the hydrophobic effect and aromatic–proline interactions (CH–π interactions)^42^, and could increase the concentration of cis-proline conformation in percentages > 20% for peptides in aqueous solution^43^.

In a subsequent step, strong and medium NOEs measurements were used as an input to derive a structure of the peptide using restrained molecular dynamics (MD). Specifically, a total of 42 strong and 148 medium no redundant NOEs were used to impose distance constrains in the interval 1.8–2.8 Å and 1.8–3.8 Å, respectively. Supplementary Table S3 lists the pairs of atoms involved in the diverse NOE measurements, grouped by their intensity. Unfortunately, simultaneous use of the 190 distance restrictions did not provide any reasonable structure, suggesting that the peptide adopts several conformations in this environment. We also proceeded to carry out diverse restrained MD simulations using different subsets of restrains that did not provide any reasonable structure either.

**Figure 5:**
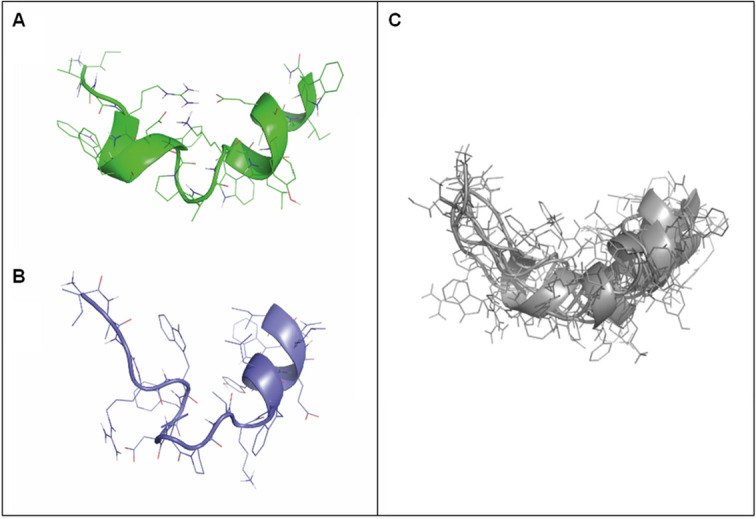
(**A**) 3D structure of the most populated conformation of the retro-enantio peptide during the sampling process with the peptide bond prior to Pro-9 in trans. (**B**) 3D structure of the most populated conformation of the peptide during the sampling process with the peptide bond prior to Pro-9 in cis. (**C**) Superimposition of several RE-E1P47 structures sampled during the MD calculation process fulfilling NOEs distance restraints. PyMOL Molecular Graphics System (v1.7.0.3.) was used.

Since the restricted MD did not provide a reasonable structure compatible with the experimental results, we proceeded to compute a 1 µs MD trajectory of the peptide unrestricted that was used to compute the values of a set of diverse atomic distances that could be contrasted with the NOEs measurements obtained from the NMR studies. In order to analyze the conformational features of the peptide along the MD trajectory, the structures sampled were clustered using the Linkage-average algorithm^44^. This analysis showed that the most populated structure (~ 85%) can be described as a helix–turn–helix similar to the structure exhibited by E1P47^25^ as shown in Fig. 5A. Analysis of a set of selected distances attained during the sampling process showed that although a majority complied with the NOE measurements (Supplementary Figures S4–S5), there were a few that did not. In order to increase the number of distance constraints fulfilled we also investigated the conformational features of the peptide when the peptide bond previous to the central proline is in cis. For this purpose we performed a short MD trajectory of the peptide at 900 K and annealed some of the structures through energy minimization. Subsequently, we selected one of the structures with the peptide bond before Pro in cis and ran a 1 µs MD simulation. After clustering the structures, a main structure (~ 35%) with a α-helical C-terminal segment and a turn at the Pro-9 (in cis) was found (Fig. 5B).

Interestingly, this structure fulfils distances 11 and 63 of the experimental NOE derived distances that were not fulfilled by the trans structure. Accordingly, we considered that the two structures coexist in solution. However, there were other NOE derived distances not fulfilled by any of the two structures. Hence, we proceeded to analyze the time evolution of those distances not satisfied by any of the two structures along the MD trajectory. These results are shown in Supplementary Figure S6. Analysis of these Figures allowed us to identify specific structures that satisfied the rest of the NOE derived distances. These conformations retained the helical structure at the C-terminus, but were more extended at the N-terminus.

Putting all this information together, the composed picture that emerged from the structural analysis carried out suggests that the peptide adopts a helix–turn–helix or an unstructured-turn–helix conformation with the peptide bond before Pro-9 in trans or in cis conformation, being the former preferred since during the 1 µs MD trajectory, the peptide bond was not isomerized. Moreover, the N-terminus appeared to be more flexible than the C-terminus (Fig. 5C). Actually, the latter was kept basically in a helical conformation, whereas the former alternated between a helix and more extended conformations.

Furthermore, peptide assembly on the membrane as well as recognition of its viral target on the membrane mimetic environment was studied by biophysical assays since it has been demonstrated that fusion inhibitor peptides specifically interfering with the N-terminal region of gp41^21^, need to be embedded into the membrane in order to interact properly with their viral target^22^.

**Figure 6:**
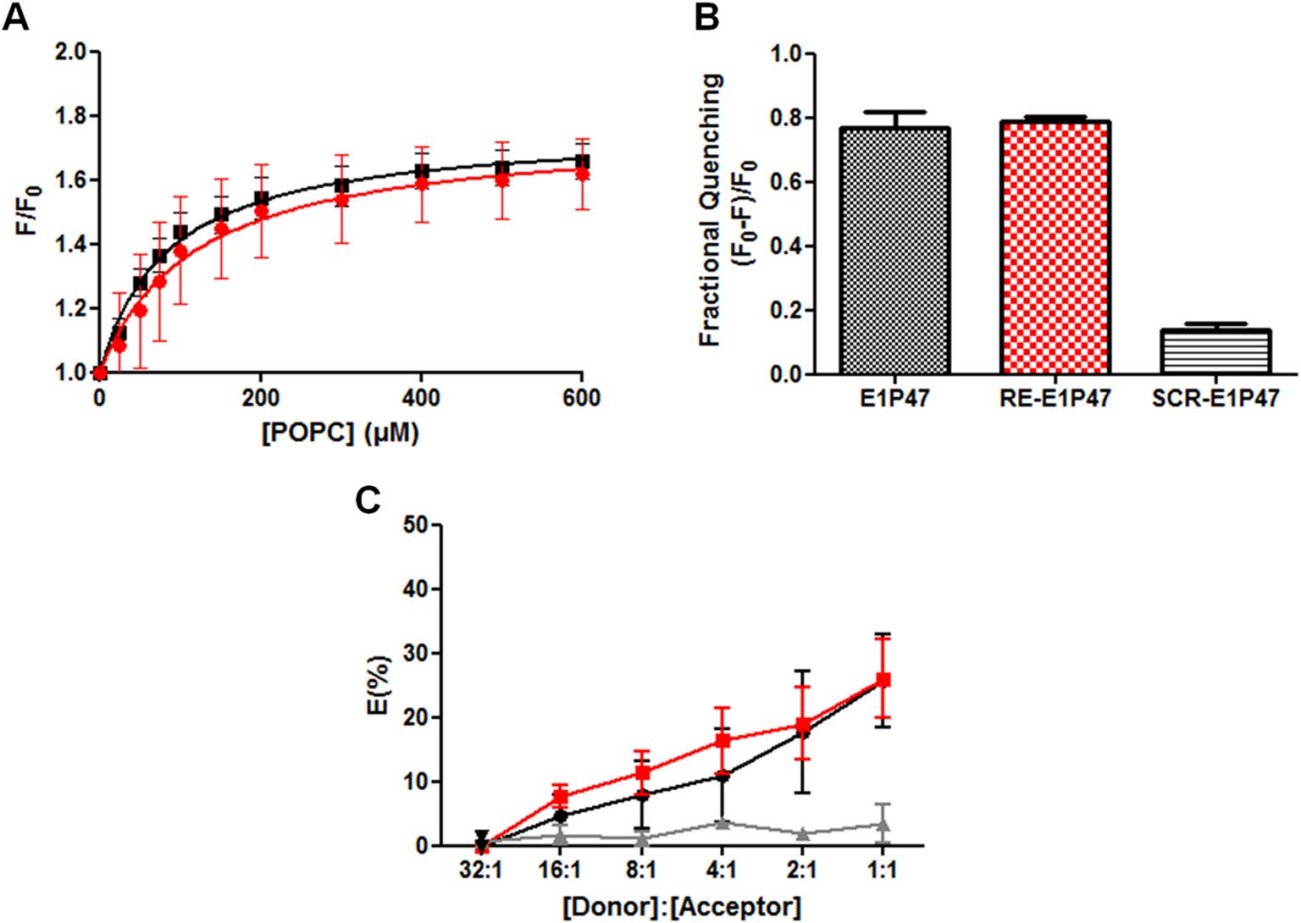
(**A**) Partitioning isotherms of E1P47 (black) and RE-E1P47 (red) estimated upon titration of 5 µM of peptides with POPC liposomes. The control peptide, SCR-E1P47, didn’t show any fluorescence increase upon POPC titration (data not shown). (**B**) Peptide Trp quenching by brominated lipid vesicles. Binding and quenching experiments were done by triplicate. (**C**) FRET efficiency between the donor (NBD-HIV-1 FP) and the acceptors (TAMRA-E1P47 in black, TAMRA-RE-E1P47 in red and TAMRA-SCR-E1P47 in grey) in presence of 100 µM of POPG liposomes. For (**A**–**C**) data shown are the means (± SEM). Software GraphPad Prism 5.0 (https://graphpad-prism.software.informer.com/5.0/) was used.

Partitioning isotherms estimated from the fractional change in Trp fluorescence intensity upon addition of increasing amounts of palmitoyl-2-oleoyl-sn-glycero-3-phosphocholine (POPC) liposomes demonstrated that the affinity of both peptides for the lipid bilayer was almost equal (Fig. 6A). In addition, collisional quenching of Trp residues by brominated lipid vesicles showed a similar accessibility of both peptides to the hydrophobic inner part of the lipid vesicles (Fig. 6B). The quenching of Trp residues in the RE-E1P47 peptide by brominated atoms at positions 9 and 10 of the hydrophobic tails of the phospholipid implied that these Trp residues point to the micelle core in almost the same manner that in L-E1P47. Thus, peptide assembly on the membrane was a requirement that the RE-E1P47 peptide fulfilled to maintain the recognition of the viral target site.

In addition, the interaction between the RE-E1P47 and HIV-1 FP peptides in a membranous environment was studied by means of Förster Resonance Energy Transfer (FRET) assay. The HIV-1 FP labelled with 6-(7-nitrobenzofurazan-4-ylamino)hexanoic acid (NBD) was the donor peptide and the RE-E1P47 labelled with 5(6)-carboxy-tetramethyl-rhodamine (TAMRA) was the acceptor peptide. As shown in Fig. 6C, the FRET percentage efficiency between the donor (NBD-HIV-1 FP) and the acceptor (TAMRA-RE-E1P47) at a 1:1 peptide concentration ratio was the same that the previously demonstrated with the acceptor (TAMRA-E1P47)^23^. A FRET control experiment was carried out between donor NBD-HIV-1 FP and acceptor TAMRA-SCR-E1P47 peptides demonstrating that there was not interaction between them.

**Figure 7:**
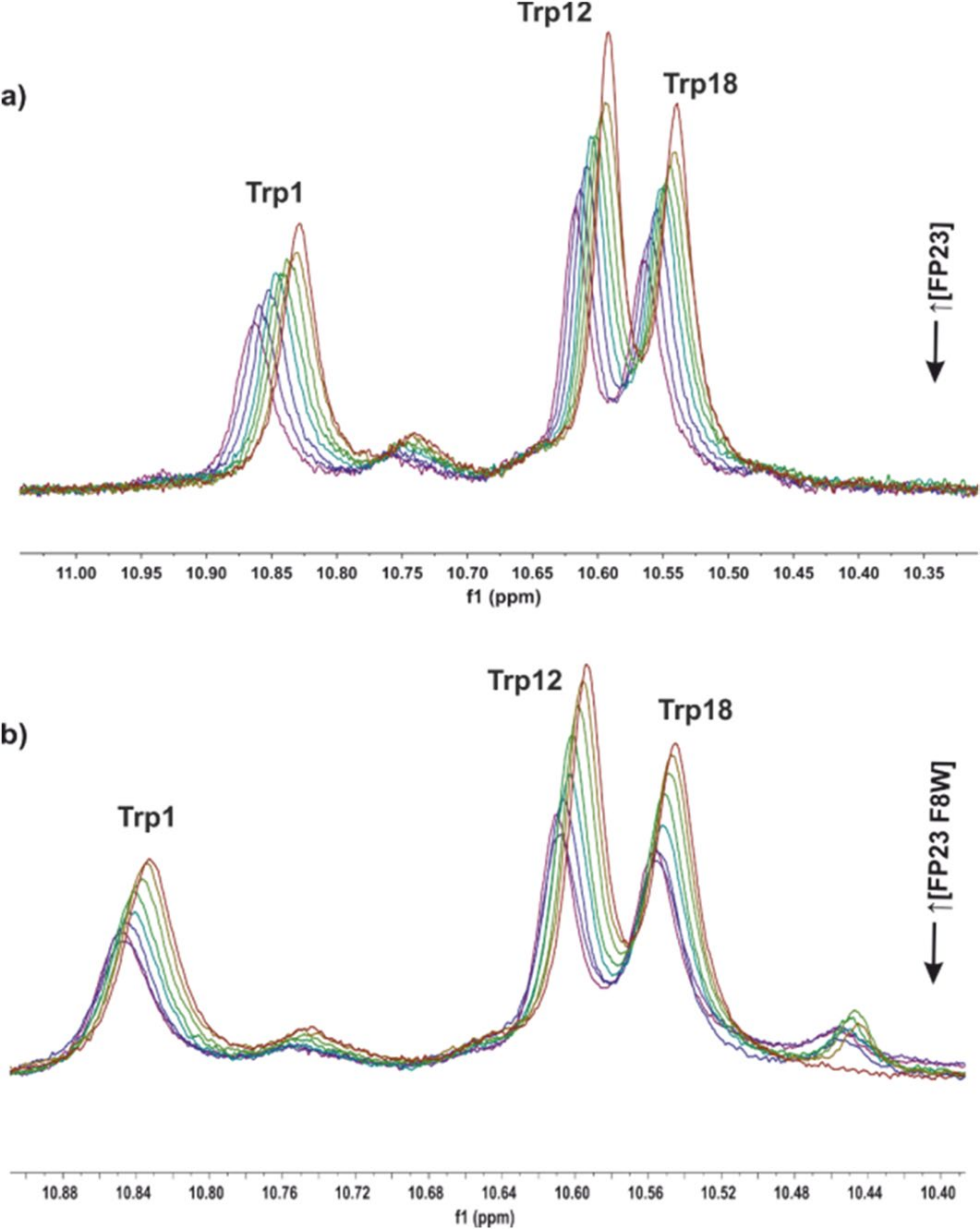
1D ^1^H NMR spectra. The association of retro-enantio RE-E1P47 with FP or FPF8W was characterized by titration of previously pre-formed RE-E1P47/DPC complexes with increasing amounts of FP dissolved in DMSO. Initially, the RE-E1P47 concentration was 0.25 mM for (**a**) and 0.39 mM for (**b**), with a 1:100 RE-E1P47:DPC ratio. FP concentrations were increased from 0.05 to 0.35 mM (0.05, 0.10, 0.15, 0.20, 0.25, 0.30 and 0.35 mM) and FPF8W from 0.09 to 0.60 mM (0.09, 0.18, 0.27, 0.35, 0.44, 0.52 and 0.60 mM). 1D ^1^H NMR spectra were acquired in 15 mM HEPES-d_11_ (90% H_2_O/10% D_2_O. pD 6.2, 303 K) using water suppression with watergate W5 pulse (zgpgw5 pulse sequence from Bruker pp library). MNova 12.0.0 software for Windows (Mestrelab Research S.L., Santiago de Compostela, Spain) was used.

To further verify this interaction, RE-E1P47–FP peptide complex formation was monitored using 1D ^1^H NMR spectra. The results showed a number of chemical shift changes during the titration. These changes were more easily followed using the Tryptophan’s NH indole protons from RE-E1P47 peptide (Figs. 7a,b) and were similar to those previously observed with L-E1P47^25^. The set of results indicated that the RE-E1P47 peptide interacted with the fusion peptide of HIV-1 gp41 similarly as the E1P47 peptide did.

On the other hand, a titration of the scrambled control peptide (SCR-E1P47) with the fusion peptide was also followed by NMR experiments and previously published^25^. The results of the titration experiment indicated that SCR-E1P47 falls off the DPC micelles after HIV-1 FP addition, perhaps due to a minor affinity for lipid micelles that HIV-1 FP and/or different location in micelles (on the surface versus inserted). The diffusion coefficients values measured corroborated these results proving that SCR-E1P47 and HIV-1 FP could not bind simultaneously to lipid micelles due to the much higher affinity of HIV-1 FP to membrane environment. This could explain the lack of inhibitory activity for SCR-E1P47.

These results led us to the assumption that the conformational flexibility of the RE-E1P47 mainly in the N-terminal end did not hinder the recognition and its interaction with the viral target and, therefore, did not compromise its anti-viral potency. Thus, RE-E1P47 was able to assemble into the membrane and subsequently to interact with the HIV-1 target similarly to the lead peptide did.

The overall results reflect the importance of designing optimized peptides from the structural knowledge of the parent peptide. The peptide affinity and its subsequent assembly into the lipid bilayer for the correct recognition of the target sites are also key issues in the design of targeted inhibitors of protein–protein interactions that take place in the cell membrane.

## Conclusions

On the basis on the structural knowledge of a previously defined HIV-1 entry inhibitor (L-E1P47), a retro-enantio and two stapled peptides have been designed to either maintain the side-chain topology or reinforce the helicity on N or C-terminus of the parent peptide. The three synthesized peptides show greater inhibitory potencies in cellular models and inhibit HIV-1 infection in colorectal tissue explants. Unlike L- and stapled E1P47 peptides, the retro-enantio analogue is highly resistant to human serum proteases, which, together with its anti-HIV-1 activity, determines its selection as optimized peptide. The structural results demonstrate a similar topology of L-E1P47 and RE-E1P47 peptides compatible with a shared helix–turn–helix conformation. Basically, the RE-E1P47 is assembled in the membrane similar to the L-E1P47 maintaining the active conformation which is required for its interaction with the viral target site.

The overall pre-clinical results demonstrated that E1P47-derivative peptides can inhibit HIV-1 infection and that sustained dosing of colorectal tissue explants with peptides results in an increase of inhibitory activity. Taking into account that HIV-1 fusion inhibitors are an important part of the repertoire of antiretroviral compounds in use and considering that RE-E1P47 targets a different region within the gp41 protein of HIV-1 than the prototype fusion T20 it constitutes the basis for further pre-clinical evaluation in animal models prior to inclusion into clinical trials.

## Methods

The work was approved by the Ethical Committee of the Consejo Superior de Investigaciones Científicas (CSIC), Madrid, Spain. All methods were performed in accordance with the relevant guidelines and regulations of IQAC-CSIC.

### Synthesis of peptides

Retro-enantiomer analog of E1P47 (RE-E1P47) was manually synthesized by solid-phase peptide synthesis (SPPS) as C- terminal carboxamide on an amino resin derivatized with the benzhydrylamine group. d-amino acids derivatives protected on N-terminus with 9-fluorenyl-methoxycarbonyl (Fmoc) were used throughout the synthesis. d-amino acid side chain protection was obtained with the following: tert-butyl (tBu) for aspartic acid, glutamic acid and tyrosine; 2,2,4,6,7-pentamethyldihydrobenzofuran-5-sulfonyl (Pbf) for arginine; and tert-butoxycarbonyl (Boc) for lysine and tryptophan. Carboxylic group of Fmoc protected D-amino acids was activated by 2-(1H-7-azabenzotriazole-1-yl)-1,1,3,3-tetramethyluronium hexafluorophosphate methanaminium (HATU) and diisopropylethylamine (DIPEA) using 3 molar excess. The Fmoc deprotection step was performed by treatment with 20% piperidine in dimethylformamide (DMF).

Once the synthesis of the peptide sequence was completed, a fraction of the peptidyl-resin was labelled at the N-terminus with 5(6)-carboxy-tetramethyl-rhodamine (TAMRA) following the procedure described in Ref.^22^.

Stapled peptides (StP1- and StP2-E1P47) were synthesized by SPPS as a C-terminal carboxamides on an ChemMatrix resin which contains the linker 5-[3,5-dimethoxy-4-(aminomethyl)phenoxy]pentanoic acid (H-PAL). l-amino acids were protected with the Fmoc group on N-terminus and almost same protecting groups used for the synthesis of the RE-E1P47 peptide. Cyclization of peptides was done on solid phase and especially protecting groups for Lys, Glu and Asp amino acids were used. Particularly, for the synthesis of StP1-E1P47 the Arg residue at position 15 was substituted for a Lys residue. For the synthesis of both stapled peptides (StP1- and StP2-E1P47), an Fmoc-Lys-OH derivative protected at Nε- with the group 4-methyltrityl (Mtt) was used as well as Fmoc-Glu-OH or Fmoc-Asp-OH derivatives protected at their side chain carboxylic group with 2-phenylisopropyl ester (2-PhiPr). The activation of the protected l-amino acids derivatives to form the peptide bond was done as described above. Once the synthesis was completed, the protecting groups Mtt and 2-PhiPr were selectively removed with 2% TFA in dichloromethane (DCM). Afterwards, cyclization was performed on solid phase by activation of the deprotected carboxylic groups of Asp12 or Glu4 with benzotriazol-1-yloxy-tripyrrolidinophosphonium hexafluorophosphate (PyBOP) and DIPEA in DMF. The reaction took place overnight with agitation to form the lactam between Asp12 and Lys14 or Glu4 and Lys8 in StP1- and StP2-E1P47, respectively.

RE-E1P47, TAMRA labelled RE-E1P47 as well as the cyclized peptidyl-resins were side-chain deprotected and cleaved from the resins by treatment with 95% TFA, 2.5% water and 2.5% TIS. After removing the TFA under N_2_ gas flow, the crude peptides were precipitated with diethyl ether and subsequently isolated.

The crude peptides were purified by RP-HPLC (Waters 1525P, Milford, MA, USA) USA) in a ZORBAX Eclipse XD8-C8 semi-preparative column (5 μm, 9.4 × 250 mm, Agilent Technologies, Santa Clara, CA, USA). Linear gradients of 30–60% of B into A in 30 min were performed with a flow of 10 ml/min. Eluent A was 0.05% TFA in water and eluent B was 0.05% TFA in acetonitrile. The peptides were 95% pure by UPLC at 220 nm.

The identity of the peptides was confirmed by electrospray ionization mass spectrometry (ES-MS) using the equipment and procedure detailed in Ref.^22^. Characterization of RE-E1P47, StP1-E1P47 and StP2-E1P47 is depicted in Figures S7–S9. The purity of all peptides was > 95%.

### Circular dichroism

CD spectra were recorded on a Jasco J-815 spectropolarimeter (Jasco International Co., Ltd., Tokyo, Japan) equipped with a Peltier type temperature controller set at 25ºC using cells of 0.1 cm in diameter. Measurements were taken using the same experimental conditions described previously^25^. Briefly, micelles of dodecylphosphorylcholine-d_38_ (DPC-d_38_) on HEPES 15 mM pH = 6.2 were used for analysing peptides (E1P47, StP1-, StP2- and RE-E1P47) at a concentration of 70 µM. The peptide:DPC ratio measured was 1:100. Three scans for each spectrum were acquired between 190 and 260 nm using a spectral bandwidth of 1 nm and a scan speed of 20 nm/min. The data were expressed in terms of mean residue ellipticities [θ] (deg cm^2^ dmol^−1^).

### NMR Spectroscopy

All data were acquired using a Bruker Avance-III 500 MHz spectrometer equipped with a z-axis pulsed field gradient triple resonance (^1^H, ^13^C, ^15^ N) TCI cryoprobe. E1P47 retro-enantio peptide samples (2 mg) were prepared by dissolving first lyophilized peptide in a small amount of DMSO-d_6_ and next, dissolution in detergent aqueous solution containing perdeuterated DPC (final molar ratio ~ 1:100). Deuterated 3-(trimethylsilyl)-1-propanesulfonic acid (DSS) (98% atom ^2^H, was added as an internal chemical shift standard for ^1^H-NMR spectroscopy and 0.05% NaN_3_ as preservative. The final pH of E1P47 peptide/detergent aqueous solution (85% H_2_0/10% D_2_0/5% DMSO-d_6_, 20 mM HEPES buffer) was adjusted to 6.0–6.2. Two-dimensional (2D) ^1^H-^1^H experiments (NOESY, ROESY, TOCSY and clean-TOCSY) spectra were acquired at 308 K in the phase-sensitive mode using the States-TPPI method and using a time domain data size of 800 t1 and 2048 t2 complex points and 64 transients per complex t1 increments. The pulses sequences used were noesyfpgpphwg, roesygpph19 and dipsi2esggph from Bruker library and modified mlevgpphw5 with additional delay for the “clean” TOCSY version. TOCSY spectra were obtained with a mixing time of 80 ms, NOESY spectra with a mixing time of 150 ms and ROESY with a mixing time of 75 ms. The comparison of NOESY and ROESY cross peaks allowed to detect those cross peaks in NOESY spectra originated by spin-diffusion. 2D homonuclear experiments were processed with the standard TOPSPIN program (Bruker Biospin, Karlsruhe, Germany). The 2D data matrices were multiplied by square-sine bell window function with the corresponding shift optimized for every spectrum and zero-filled to 2 × 1 K complex matrices prior to Fourier transformation. Baseline correction was applied in both dimensions. Peptide NMR resonances were assigned by analysing the 2D NMR spectra using the CCPN analysis 2.3 software (available from the Collaborative Computing Project for NMR, www.ccpn.ac.uk) following the standard sequential assignment strategy^45^. NOE cross-peaks were integrated in the 150 ms NOESY spectra and the NOE volumes were converted to distances, which were calibrated using the average NOE volume of resolved geminal methylene proton cross-peaks from Asp-12. According to the procedure of Wuthrich et al., each distance was converted to a distance restraint by calculating upper distance bounds and was classified into strong (1.8–2.8 Å), medium (1.8–3.8 Å), weak (1.8–5.0 Å) and very weak. The pseudoatom corrections were applied to NOE constraints involving equivalent or non-stereo assigned^46^.

### Molecular modelling studies

The starting structure of the E1P47 retro-enantio peptide was generated with the MOE program 40 as an extended conformation of the peptide with its N-terminus charged and the C-terminus amidated. Molecular dynamics (MD) simulations were carried out with the AMBER14 software 41 using the force field ff99SB at 300 K using the NVT collective. Simulations were carried out in implicit solvent using a Generalized Born model^47^ with the dielectric constant of methanol (ε = 32.7). Moreover, a 2 fs integration step was used for the molecular dynamics runs after constraining all the bonds involving hydrogen atoms using the SHAKE algorithm^48^. Before starting the molecular dynamics calculations the structure was energy minimized using the steepest descent method. After minimization, the system was heated to 300 K using a molecular dynamics calculation within the NVT collective at a rate of 30 K per 10 ps. Subsequently, several 10 ns restrained molecular dynamics simulations were performed using distance restrains deduced from 41 strong and 144 medium NOEs signals, imposing a 1.8–2.8 Å and 2.8–3.8 Å, respectively with a force constant of 20 kcal mol^−1^ Å^−2^ to the corresponding atoms involved. In a following step, we carried out 1 μs MD trajectory at 300 K of the peptide using no restrains. A 10 ns MD simulation at 900 K allowed us to identify a conformation of the E1P47 retro-enantio peptide with the peptide bond previous to Pro in cis. This conformation was minimized and used as starting point of a new 1 μs MD trajectory at 300 K. Subsequently, atoms distances involved in the NOE signals were computed in the structures obtained from the 1 μs MD trajectories and compared with the experimental NOEs obtained from NMR studies. The structures sampled during the MD trajectory were clustered using linkage-average hierarchical clustering method^49^.

### Fluorescence assays

Large unilamellar vesicles (LUV) composed of different phospholipids were prepared as model membranes as described previously^50^. Membrane partition studies with E1P47, RE-E1P47 and a scrambled peptide (SCR-E1P47) were carried out in a PTI Fluorescence Master Systems spectrofluorimeter (Photon Technology International, Birmingham, AL, USA). The scrambled peptide (SRC-E1P47: ELDIRLKGFFVWIVWPWY) was selected as a negative control^25^. Emission fluorescence spectra were recorded for E1P47, RE-E1P47, SCR-E1P47 in PBS (0.01 M, pH 7.4) at 20ºC using an excitation wavelength of 280 nm. Changes in the Trp fluorescence spectra were monitored after incubation of 5 µm of each peptide with increasing concentrations of POPC liposomes as described in Ref.^22^.

Quenching of Trp by brominated phospholipids was performed using liposomes composed of 1,2-di-(9-10-dibromo) stearoyl-sn-glycero-3-phosphocoline (Br-PC). Comparatively, a concentration of 2 µM of each peptide (E1P47, RE-E1P47 and SCR-E1P47) was added to 200 µM of lipid vesicles either BrPC or 1,2-dioleoyl-sn-glycero-3-phosphocholine (DOPC). The Trp fluorescence was recorded with an excitation wavelength of 290 nm and emission from 300 to 450 nm and the fractional quenching of Trp fluorescence was calculated as described in Ref.^22^.

To perform Fluorescence Resonance Energy Transfer (FRET) assays, NBD-labelled HIV-1 FP and TAMRA-labelled E1P47, RE-E1P47 and SCR-E1P47 peptides were used as donor and acceptors, respectively. NBD-HIV-1 FP and TAMRA-E1P47 peptides were previously synthesized^23^. The emission spectra were recorded in the wavelength range 450–650 nm upon excitation at λ = 467 nm. NBD HIV-1 FP was added to a 100 µM suspension of palmitoyl-2-oleoyl-sn-glycero-3-phosphoglycerol (POPG) liposomes to reach a final concentration of 0.4 µM. Comparatively, TAMRA-labelled E1P47 and RE-E1P47 peptides in doses ranging from 0.0125 to 0.4 µM were sequentially added to the LUV solutions containing the NBD-HIV-1 FP. The percentage efficiency of the energy transfer was determined from the ratio of the donor in the presence and the absence of the acceptor as described in Ref.^23^.

### Stability in human serum

The stability of E1P47 peptides was studied in human serum, a protease-rich media. Briefly, a concentration of 1 mg/ml of each peptide was incubated with 90% human serum in Hanks Balanced Salt Solution (HBSS) at 37ºC and aliquots of 40 µl were taken at different time-points during 24 h and treated with 200 µl of methanol to immediately precipitate the serum proteins. After 30 min of centrifugation at 4 °C, the supernatant was analysed by HPLC at 220 nm to calculate the content of intact peptide in each sample and by UPLC/MS to identify the main metabolites generated after the enzymatic hydrolysis.

### HIV-1 inhibition assays in ex vivo human tissue explants

#### Virus

HIV-1_BaL_^51^ was provided by the NIH AIDS Research & Reference Reagent Program (https://www.aidsreagent.org/). Viral stock was prepared by passage through activated PBMCs^52^ for 11 days.

#### Patients and tissue explants

Surgically-resected specimens of colorectal tissue were collected at St George’s Hospital, London and St Mary’s Hospital, Imperial College London, UK. All tissues were collected after receiving signed informed consent from all patients through the Imperial College Healthcare Tissue Bank approved by Research Ethics Committee Wales (IRAS 17/WA/0161). All patients were HIV negative. On arrival in the laboratory, resected tissue was cut into 2–3 mm^3^ explants comprising both epithelial and muscularis mucosae as described previously^36^. Colorectal explants were maintained with DMEM containing 10% fetal calf serum, 2 mM l-glutamine and antibiotics (100 U of penicillin/ml, 100 µg of streptomycin /ml, 80 µg of gentamicin /ml) at 37 °C in an atmosphere containing 5% CO_2_.

#### Infectivity and inhibition assays

Inhibition assays in tissue explants models were performed using a standardized amount of virus culture supernatant normalized for infectivity. Tissue explants were incubated with serial dilutions of peptides for 1 h before virus (10^3^ TCID_50_) was added for 2 h. Explants were then washed 4 times with PBS to remove unbound virus and drug. Colorectal explants were then transferred onto gelfoam rafts (Welbeck Pharmaceuticals, UK) and cultured for 15 days as previously described^53^ in the presence (mimicking sustained exposure) or absence (mimicking pulse exposure) of drug. Approximately 50% of the supernatants were harvested every 2–3 days and explants were re-fed with fresh media. The extent of virus replication in tissue explants was determined by measuring the p24 antigen concentration in supernatants (HIV-1 p24 ELISA, Zeptometrix Corporation, Buffalo, NY).

#### Statistical and mathematical analysis

IC_50_ values were calculated from sigmoid curve fitted (Prism, GraphPad) fulfilling the criterion of R^2^ > 0.7.

## Supplementary information

Supplementary Information
