# Supplementary material for: Importance of structure-based studies for the design of a novel HIV-1 inhibitor peptide
Source: Sci Rep. 2020 Sep 2;10:14430. doi: 10.1038/s41598-020-71404-0 (PMC7468280; doi:10.1038/s41598-020-71404-0)
Supplement: Supplementary file 1 — Supplementary Information [file 41598_2020_71404_MOESM1_ESM.pdf]

**SUPPLEMENTARY INFORMATION**

# IMPORTANCE OF STRUCTURE-BASED STUDIES FOR THE DESIGN OF A NOVEL HIV-1 INHIBITOR PEPTIDE

María José Gómara,[a] Yolanda Pérez,[b] Patricia Gómez-Gutiérrez,[c] Carolina Herrera,[d] Paul Ziprin,[e], Javier P. Martínez, [f] Andreas Meyerhans,[f,g] Juan Jesús Pérez, [c] and Isabel Haro,*[a]

[a] Unit of Synthesis and Biomedical Applications of Peptides, IQAC-CSIC. Jordi Girona, 18-26, 08034, Barcelona, Spain

[b] Nuclear Magnetic Resonance Facility, IQAC-CSIC. Jordi Girona, 18-26, 08034, Barcelona, Spain

[c] Department of Chemical Engineering (ETSEIB). Universitat Politècnica de Catalunya. Barcelona, Spain

[d] Department of Medicine, Imperial College London. London, UK

[e] Department of Surgery and Cancer, St. Mary's Hospital, Imperial College London. London, UK

[f] Infection Biology Laboratory, Department of Experimental and Health Sciences, Universitat Pompeu Fabra, Barcelona, Spain.

[g] ICREA, Pg. Lluís Companys 23, 08010 Barcelona, Spain

**Table S1**. Predicted protease cleavage sites of E1P47 peptide (WILEYLWKVPFDFWRGVI)

| Protease name | Position | P4-P4’ site | N-fragment (kDa) | C-fragment (kDa) | Cleavage Score |
|---|---|---|---|---|---|
| Cathepsin K | 4 | WILE / YLWK | 0.54 | 1.92 | 1.04 |
| Matrix metallopeptidase-9 | 16 | FWRG / VI | 2.25 | 0.21 | 1.04 |
| Matrix metallopeptidase-9 | 12 | VPFD / FWRG | 1.58 | 0.87 | 1.03 |
| Matrix metallopeptidase-3 | 12 | VPFD / FWRG | 1.58 | 0.87 | 0.96 |
| Chymotrypsin A (cattle-type) | 11 | KVPF / DFWR | 1.47 | 0.98 | 1.06 |
| Chymotrypsin A (cattle-type) | 1 | W / ILEY | 0.19 | 2.27 | 0.95 |
| Cathepsin G | 7 | EYLW / KVPF | 1.00 | 1.46 | 1.00 |

**Table S2**. Resonance assignments for RE-E1P47 peptide in DPC-d38 micelles.

| | HN | HA | HB | HB´ | HG | HG´ | HD | HD´ | HE | Others |
|---|---|---|---|---|---|---|---|---|---|---|
| Ile1 | | | | | | | | | | |
| Val2 | | 3.89 | 1.43 | | 0.93 | | | | | |
| Gly3 | 8.32 | | | | | | | | | |
| Arg4 | 7.60 | 4.66 | 1.43 | | 1.35 | | 3.08 | | | |
| Trp5 | 8.19 | 4.30 | 3.17 | | | | | | | 7.39 (HD1), 10.19 (HE1), 7.47 (HE3), 7.42 (HZ2), 6.95 (HZ3), 7.07 (HH2) |
| Phe6 | 7.75 | 4.47 | 2.25 | 2.82 | | | | | | 6.80 (HD), 7.21 (HE), 7.14 (HZ) |
| Asp7 | 8.14 | 4.54 | 2.57 | | | | | | | |
| Phe8 | 8.63 | 3.64 | 3.00 | 3.41 | | | | | | 7.22 (HD), 7.29 (HE), 7.26 (HZ) |
| Pro9 | | | | | | | | | | |
| Val10 | 8.03 | 3.53 | 1.18 | | -0.30 | 0.57 | | | | |
| | 7.34 | 3.83 | 2.19 | | 0.60 | 1.09 | | | | |
| | | 3.88 | 2.20 | | 0.69 | 1.00 | | | | |
| Lys11 | 8.23 | 4.00 | 1.92 | | 1.46 | | 1.52 | | 2.71 | 7.55 (NHZ) |
| Trp12 | 7.91 | 4.40 | 2.47 | 3.03 | | | | | | 7.22 (HD1), 10.55 (HE1), 7.44 (HE3), 7.34 (HZ2), 6.84 (HZ3), 6.99 (HH2) |
| Leu13 | 7.96 | 3.83 | 1.71 | 1.82 | 1.65 | | 0.90 | | | |
| | | 4.11 | 1.71 | 1.82 | 1.65 | | 0.90 | | | |
| Tyr14 | 8.37 | 4.28 | 3.16 | 3.36 | | | | | | 7.01 (HD), 6.60 (HE), |
| Glu15 | 8.31 | 3.83 | 2.41 | 2.62 | 2.04 | 2.17 | | | | |
| Leu16 | 7.70 | 3.89 | 1.43 | 1.40 | | | 0.56 | | | |
| Ile17 | 8.43 | 3.89 | 1.93 | | 1.19 | 1.53 | | 0.86 | | 0,94 (HG2) |
| Trp18 | 8.17 | 4.35 | 3.19 | 3.34 | | | | | | 7.29 (HD1), 10.52 (HE1), 7.43 (HE3), 7.43 (HZ2), 6.88 (HZ3), 7.02 (HH2) |

**Table S3**. Strong and medium NOE distance restraints.

Strong NOEs:

```
distance  d1        :5@H,                    :2@HA *1
distance  d2        :5@H,                    :2@HB
distance  d3        :5@HD1,                  :5@HE1
distance  d4        :5@HZ2,                  :7@HA *1
distance  d5        :6@H,                    :6@HA
distance  d6        :6@HE1,HE2,              :6@HD1,HD2
distance  d7        :7@H,                    :6@HA
distance  d8        :8@HZ,                   :7@HA *1
distance  d9        :10@HA,                  :10@HB
distance  d10       :10@HB,                  :12@HE3
distance  d11       :10@HB,                  :12@HZ3
distance  d12       :11@HB2,                 :11@HB3
distance  d13       :12@H,                   :14@HA *1
distance  d14       :12@HB2,                 :12@HB3
distance  d15       :12@HD1,                 :12@HB3
distance  d16       :12@HE1,                 :12@HD1
distance  d17       :12@HE3,                 :14@HA
distance  d18       :12@HH2,                 :12@HZ2
distance  d19       :12@HH2,                 :12@HZ3
distance  d20       :12@HZ2,                 :12@HE1
distance  d21       :12@HZ3,                 :14@HA
distance  d22       :13@HA,                  :12@HD1
distance  d23       :13@HG,                  :13@HB3
distance  d24       :14@H,                   :15@H
distance  d25       :14@HB3,                 :12@HD1
distance  d26       :14@HB3,                 :14@HB2
distance  d27       :15@H,                   :14@HA
distance  d28       :15@H,                   :14@HB3
distance  d29       :15@H,                   :18@HA
distance  d30       :15@HA,                  :15@H
distance  d31       :15@HB3,                 :15@HB2
distance  d32       :15@HG3,                 :18@HA *1
distance  d33       :16@HB2,                 :16@HB3
distance  d34       :17@H,                   :13@HA
distance  d35       :17@H,                   :15@HA
distance  d36      :18@HD1,                 :18@HE1
distance  d37       :18@HE3,                 :18@HB2
distance  d38       :18@HH2,                 :18@HZ2
distance  d39       :18@HH2,                 :18@HZ3
distance  d40       :18@HZ2,                 :18@HE1
distance  d41       :18@HZ3,                 :18@HE3
```

Medium NOEs:

```
distance  d42       :3@H,                    :2@HA
distance  d43       :3@H,                    :5@HD1
distance  d44       :3@H,                    :6@H
distance  d45       :4@H,                    :5@HA
distance  d46       :4@H,                    :5@HZ3
distance  d47       :5@H,                    :4@HB2,HB3
distance  d48       :5@H,                    :5@HB2,HB3
distance  d49       :5@HA,                   :6@HB3
distance  d50       :5@HD1,                  :2@HA
distance  d51       :5@HD1,                  :5@HB2,HB3
distance  d52       :5@HZ3,                  :5@HE3
distance  d53       :6@H,                    :7@HB2,HB3
distance  d54       :6@HA,                   :10@HB
distance  d55       :6@HA,                   :7@HB2,HB3
distance  d56       :6@HB2,                  :6@HB3
distance  d57       :6@HB3,                  :6@H
distance  d58       :6@HB3,                  :6@HD1,HD2
distance  d59       :7@H,                    :5@HB2,HB3
distance  d60       :7@H,                    :6@H
distance  d61       :7@H,                    :7@HB2,HB3
```

```
distance  d62     :7@HA,                 :9@HB2,HB3
distance  d63     :8@HA,                 :11@HB2
distance  d64     :8@HA,                 :7@H
distance  d65     :8@HB2,                :7@H
distance  d66     :8@HB2,                :8@HA
distance  d67     :8@HB3,                :7@H
distance  d68     :8@HB3,                :8@HA
distance  d69     :8@HB3,                :8@HD1,HD2
distance  d70     :8@HZ,                 :13@HG
distance  d71     :9@HB2,                :18@H
distance  d72     :10@H,                 :11@H
distance  d73     :10@H,                 :12@H
distance  d74     :10@H,                 :12@H
distance  d75     :10@H,                 :17@HB
distance  d76     :10@H,                 :9@HB2
distance  d77     :10@HA,                :10@H
distance  d78     :10@HA,                :10@H
distance  d79     :10@HA,                :10@H
distance  d80_1   :10@HA,                :10@HG11,HG12,HG13
distance  d80_2   :10@HA,                :10@HG21,HG22,HG23
distance  d81     :10@HA,                :11@H
distance  d82     :10@HA,                :11@HG2,HG3
distance  d83     :10@HA,                :12@H
distance  d84     :10@HA,                :4@HG2
distance  d85     :10@HA,                :6@HZ
distance  d86     :10@HB,                :10@H
distance  d87     :10@HB,                :10@HA
distance  d88     :10@HB,                :12@H
distance  d89_1   :10@HG11,HG12,HG13     :5@HZ2
distance  d89_2   :10@HG21,HG22,HG23     :5@HZ2
distance  d90     :11@H,                 :10@HB
distance  d91     :11@H,                 :11@HA
distance  d92     :11@HA,                :11@HB2,HB3
distance  d93     :11@HA,                :11@HD2
distance  d94     :11@HA,                :14@H
distance  d95     :11@HA,                :14@HB2
distance  d96     :11@HA,                :14@HB2
distance  d97     :11@HA,                :14@HB3
distance  d98    :11@HA,                 :14@HB3
distance  d99    :11@HA,                 :15@H
distance  d100    :11@HA,                :18@H *2
distance  d101    :12@H,                 :12@HB3
distance  d102    :12@H,                 :18@HE3
distance  d103    :12@H,                 :18@HZ2
distance  d104    :12@H,                 :6@HZ
distance  d105    :12@HB2,               :12@H
distance  d106    :12@HB2,               :12@HD1
distance  d107    :12@HD1,               :10@HB
distance  d108    :12@HE3,               :12@HB2
distance  d109    :13@H,                 :12@H
distance  d110    :13@H,                 :12@HA
distance  d111    :13@H,                 :18@HB2
distance  d112    :13@HA,                :12@H
distance  d113    :13@HA,                :13@H
distance  d114    :13@HA,                :13@HG
distance  d115    :13@HA,                :14@H
distance  d116    :13@HA,                :15@HG2
distance  d117    :13@HA,                :18@HE3
distance  d118    :13@HA,                :18@HZ2
distance  d119    :13@HA,                :9@HB2,HB3
distance  d120    :13@HB2,               :14@HA
distance  d121    :13@HB2,               :15@HA *2
distance  d122    :13@HB3,               :12@HE3
distance  d123    :13@HB3,               :13@H
distance  d124    :13@HB3,               :14@H
distance  d125    :13@HB3,               :14@HB2
distance  d126    :13@HB3,               :14@HB3
distance  d127    :13@HB3,               :18@H
```

| | | | |
|---|---|---|---|
| distance | d128 | :13@HB3, | :18@HZ3 |
| distance | d129 | :13@HG, | :11@HA |
| distance | d130 | :13@HG, | :11@HB2,HB3 |
| distance | d131 | :13@HG, | :12@HD1 |
| distance | d132 | :14@H, | :13@H |
| distance | d133 | :14@H, | :14@HB2 |
| distance | d134 | :14@H, | :14@HB3 |
| distance | d135 | :14@HD1,HD2, | :12@H $*_2$ |
| distance | d136 | :14@HD1,HD2, | :14@HB2 |
| distance | d137 | :14@HD1,HD2, | :14@HB3 |
| distance | d138 | :15@H, | :12@HD1 |
| distance | d139 | :15@H, | :13@H |
| distance | d140 | :15@H, | :14@HB2 |
| distance | d141 | :15@H, | :15@HB2 |
| distance | d142 | :15@H, | :16@H |
| distance | d143 | :15@H, | :18@H |
| distance | d144 | :15@HA, | :14@HB2 |
| distance | d145 | :15@HA, | :14@HB3 |
| distance | d146 | :15@HA, | :15@HB2 |
| distance | d147 | :15@HA, | :15@HB3 |
| distance | d148 | :15@HA, | :16@H |
| distance | d149 | :15@HA, | :18@HE1 |
| distance | d150 | :15@HB2, | :15@HG2 |
| distance | d151 | :15@HB3, | :15@H |
| distance | d152 | :15@HB3, | :15@HG2 |
| distance | d153 | :15@HB3, | :15@HG3 |
| distance | d154 | :15@HB3, | :16@H |
| distance | d155 | :15@HG3, | :15@H |
| distance | d156 | :16@H, | :12@H |
| distance | d157 | :16@H, | :12@HD1 |
| distance | d158 | :16@H, | :12@HZ2 |
| distance | d159 | :16@H, | :14@HA |
| distance | d160 | :16@H, | :14@HD1,HD2 |
| distance | d161 | :16@H, | :16@HB2 |
| distance | d162 | :16@HA, | :16@HB2,HB3 |
| distance | d163 | :16@HA, | :16@HB2,HB3 |
| distance | d164 | :16@HG, | :12@HZ2 |
| distance | d165 | :16@HG, | :15@HA |
| distance | d166 | :17@H, | :14@HB3 |
| distance | d167 | :17@H, | :15@H |
| distance | d168 | :17@H, | :18@H |
| distance | d169 | :17@HB, | :15@HA |
| distance | d170 | :17@HB, | :18@H |
| distance | d171 | :17@HB, | :18@HD1 |
| distance | d172 | :17@HG12, | :15@HA |
| distance | d173 | :17@HG12, | :16@H |
| distance | d174 | :17@HG12, | :17@HD11,HD12,HD13 |
| distance | d175 | :17@HG21,HG23, | :17@HB |
| distance | d176 | :18@H, | :13@H $*_2$ |
| distance | d177 | :18@H, | :18@HB2 |
| distance | d178 | :18@H, | :18@HB3 |
| distance | d179 | :18@HB3, | :18@HE3 |
| distance | d180 | :18@HD1, | :15@HG2 |
| distance | d181 | :18@HD1, | :15@HG3 |
| distance | d182 | :18@HD1, | :16@H |
| distance | d183 | :18@HD1, | :18@HB2 |
| distance | d184 | :18@HD1, | :18@HB3 |
| distance | d185 | :18@HZ3, | :15@HA |

$*_1$ NOE in the limit strong/medium
$*_2$ NOE in the limit medium/weak

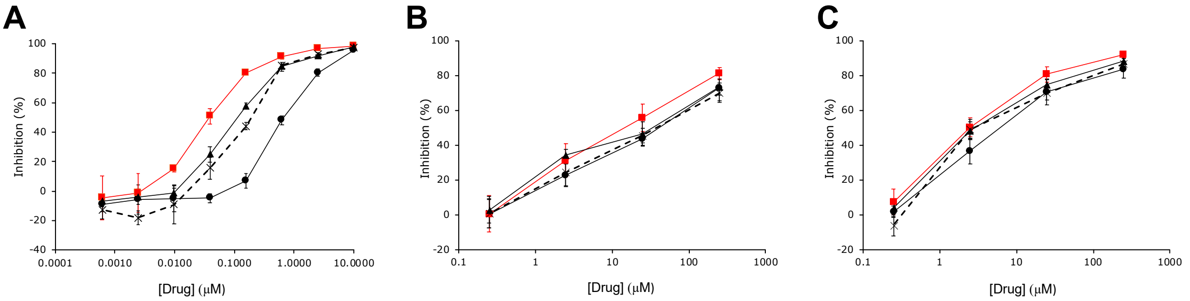


**Figure S1**. Inhibitory activity of E1P47 peptide and derivatives against HIV-1$_{BaL}$ infection. TZM-bl cells (A) and colorectal tissue explants (B, C) were treated for 1 h in the presence or absence of E1P47 (●), RE- E1P47 (■), StP1- E1P47 (▲), or StP2- E1P47 (×) prior to addition of HIV-1$_{BaL}$. Luciferase expression in TZM-bl cells (measured in relative light units) was determined after 48 h of culture in the presence of drug (sustained exposure), and the extent of inhibition by each drug was calculated. Tissue explants were washed four times with PBS after 2 h of incubation with virus and then transferred to gelfoam rafts. Explants were kept in culture for 15 days in the (B) absence (pulse exposure) or (C) presence (sustained exposure) of peptide. The concentrations of p24 in the harvested supernatants were quantified by ELISA at days 11 and 15 of culture, and the extent of inhibition by each compound at each time point was calculated. The percentage of inhibition was normalized relative to the relative light units obtained for TZM-bl cells or to the p24 values obtained for tissue explants not exposed to virus (0% infectivity) and for cells or explants infected with virus in the absence of compound (100% infectivity). Data are the means (± SEM) from three independent experiments performed in triplicate.

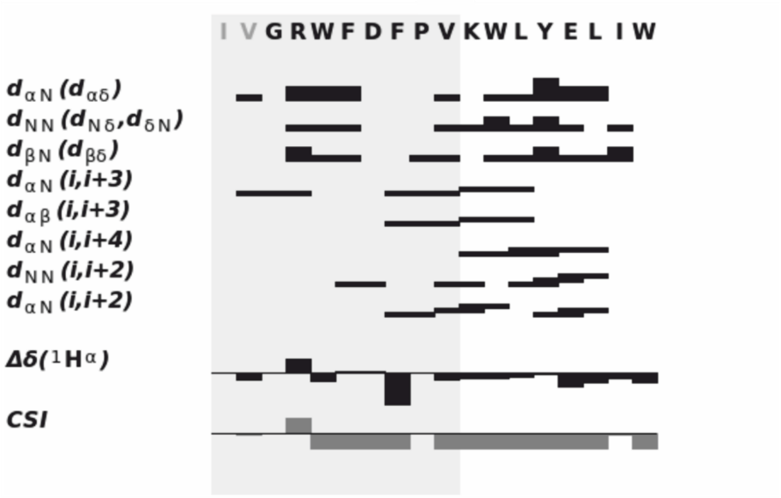


**Figure S2**. Structural Analysis of RE-E1P47 peptide. NOE summary, Chemical Shift Index (CSI) and hydrogen alpha secondary chemical shift (difference to random coil value, $\Delta\delta(^1H\alpha)$) are shown for RE-E1P47 major species in 100 mM DPC-$d_{38}$, pD 6.2. The relative intensity of the NOEs is represented by the thickness of the bars. Random coil $^1H\alpha$ values were taken from Ref. (Wishart, D.S. et al. $^1H$, $^{13}C$ and $^{15}N$ random coil NMR chemical shifts of the common amino acids. I. Investigations of nearest-neighbor effects. *J. Biomol. NMR* **5**, 67-81, 1995).

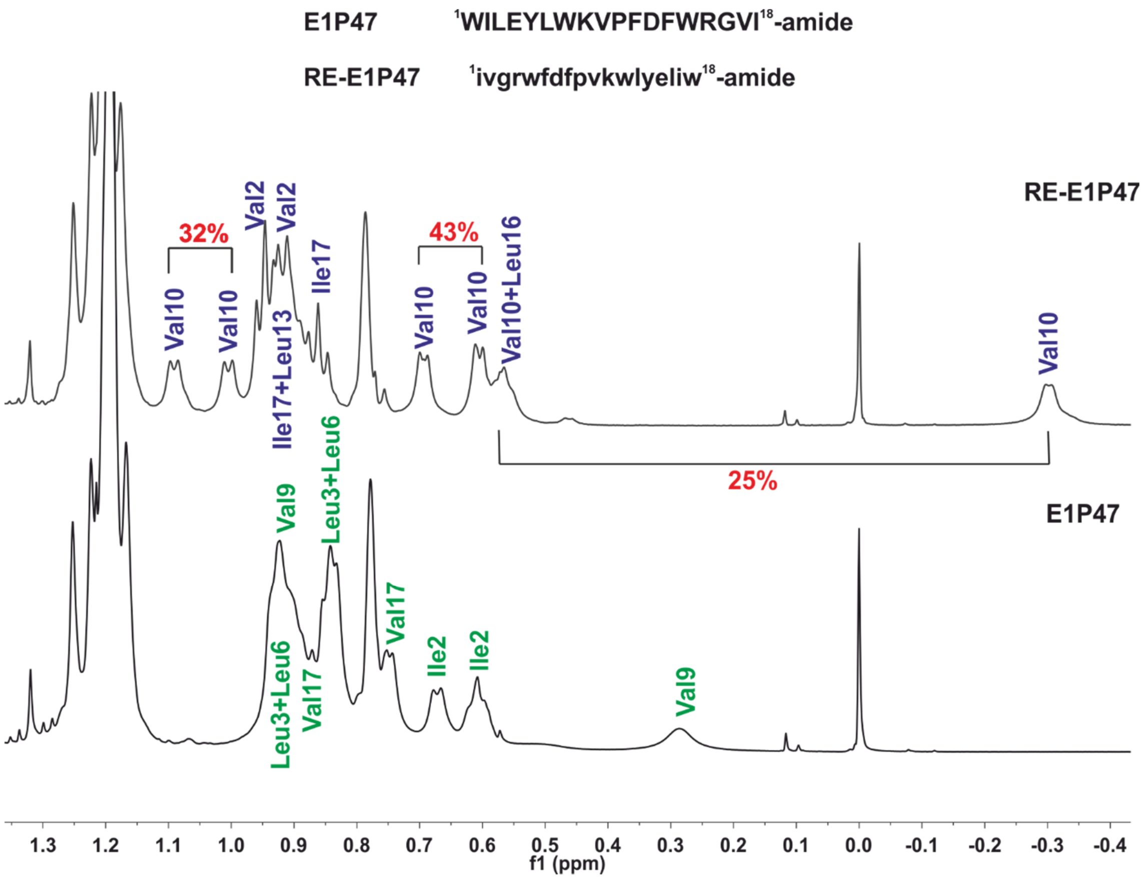


**Figure S3**. Aliphatic region of $^1H$ spectra of E1P47 (bottom) and its retro enantio version (top) in 100:1 DPC-$d_{38}$:peptide, in 15 mM HEPES-$d_{11}$, 90% $H_2O$/10% $D_2O$, pH* 6.2, at 308K. The relative percentage (calculated using peak integration) for the three populations of Val-10 are indicates in red.

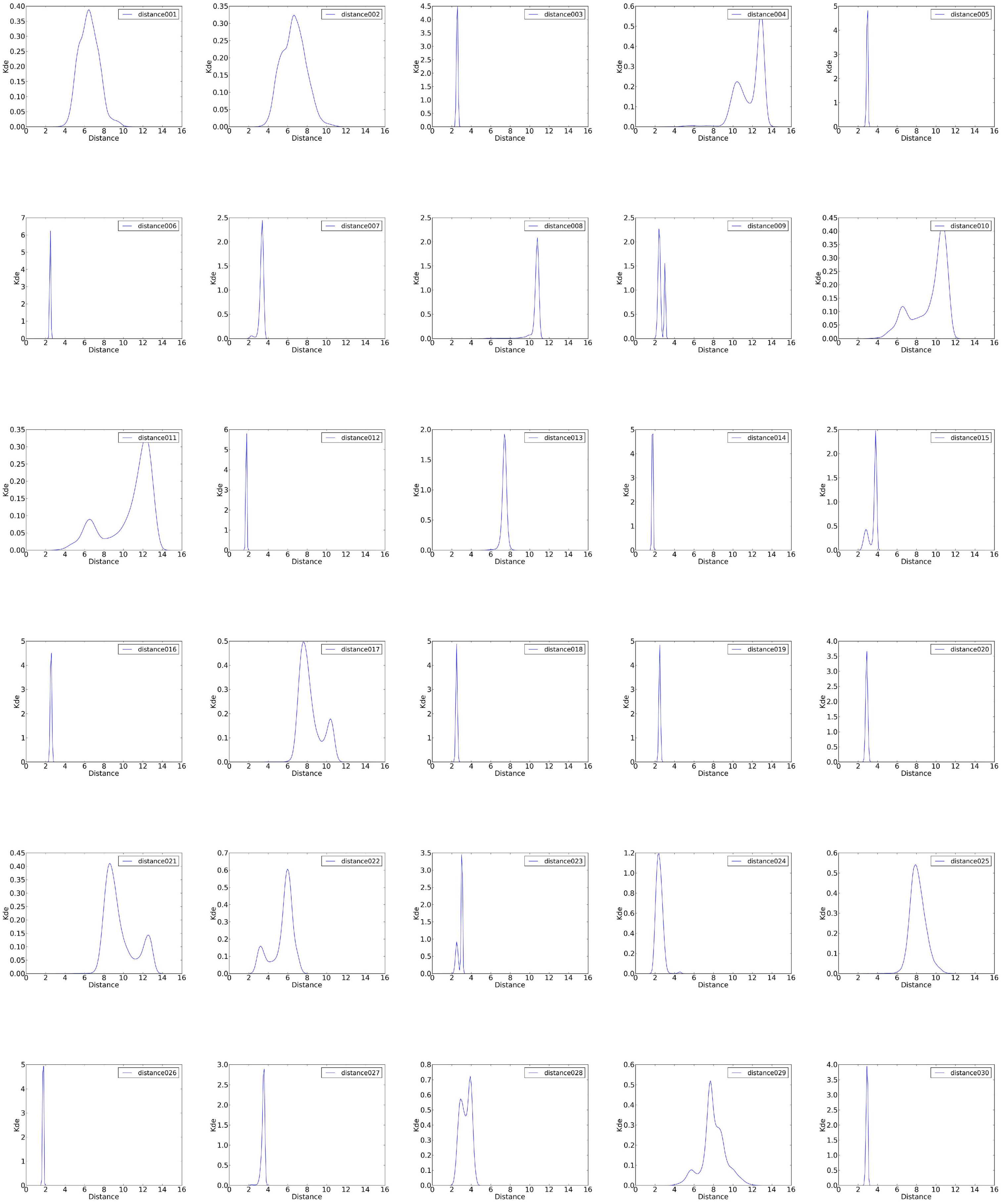
distance001
distance002
distance003
distance004
distance005
distance006
distance007
distance008
distance009
distance010
distance011
distance012
distance013
distance014
distance015
distance016
distance017
distance018
distance019
distance020
distance021
distance022
distance023
distance024
distance025
distance026
distance027
distance028
distance029
distance030
Kde
Distance

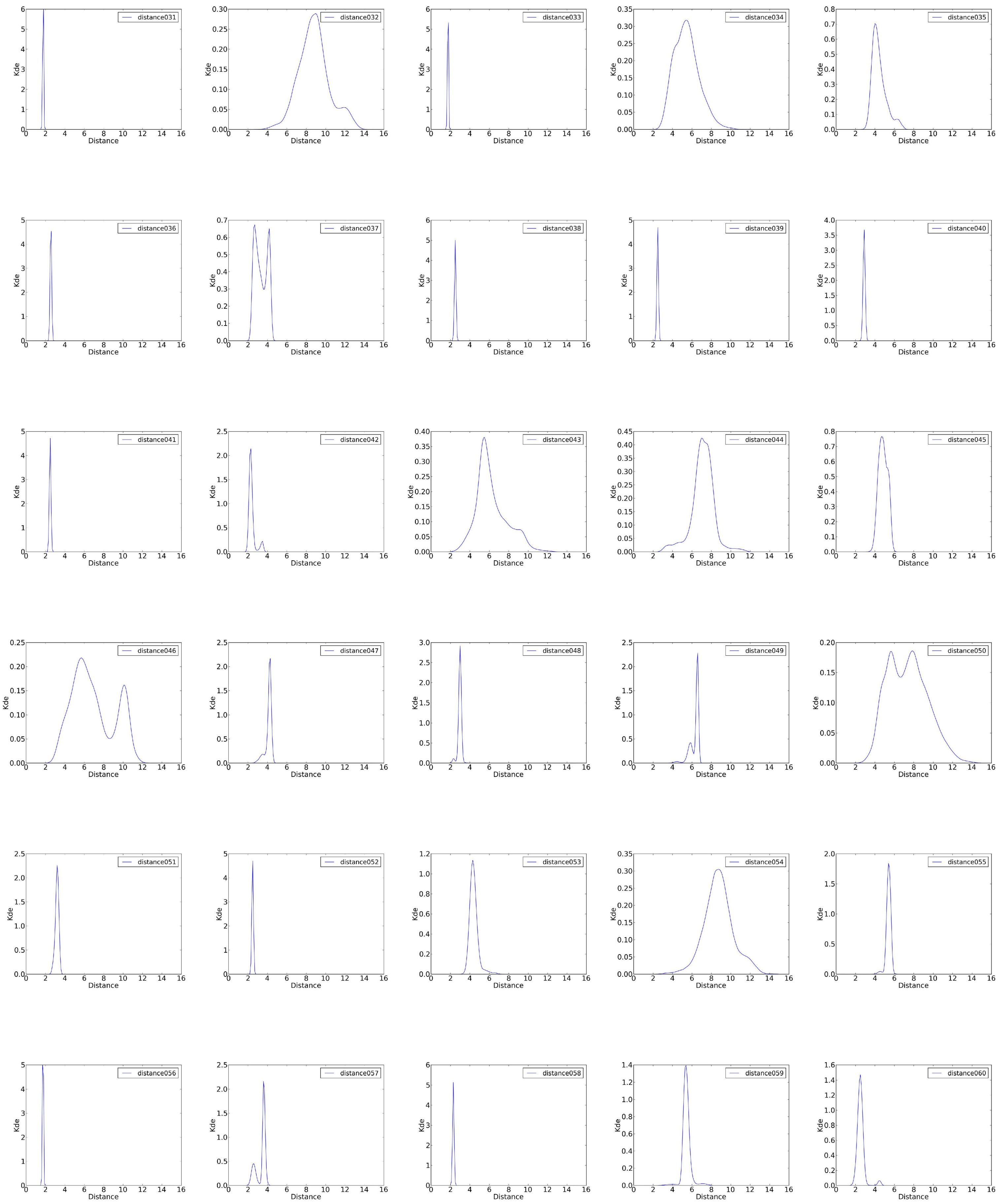
distance031
distance032
distance033
distance034
distance035
distance036
distance037
distance038
distance039
distance040
distance041
distance042
distance043
distance044
distance045
distance046
distance047
distance048
distance049
distance050
distance051
distance052
distance053
distance054
distance055
distance056
distance057
distance058
distance059
distance060
Kde
Distance

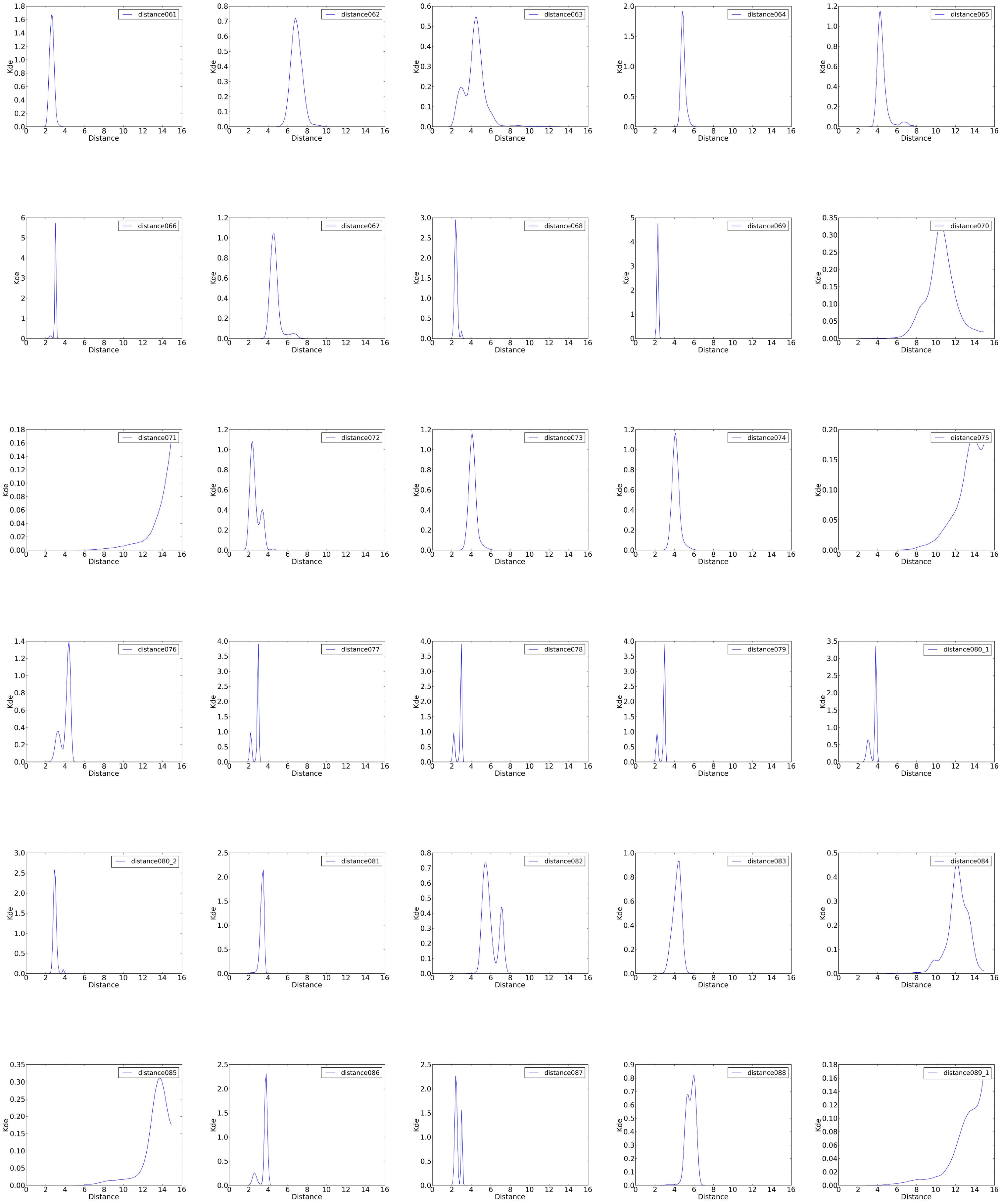
distance061
distance062
distance063
distance064
distance065
distance066
distance067
distance068
distance069
distance070
distance071
distance072
distance073
distance074
distance075
distance076
distance077
distance078
distance079
distance080_1
distance080_2
distance081
distance082
distance083
distance084
distance085
distance086
distance087
distance088
distance089_1
Kde
Distance

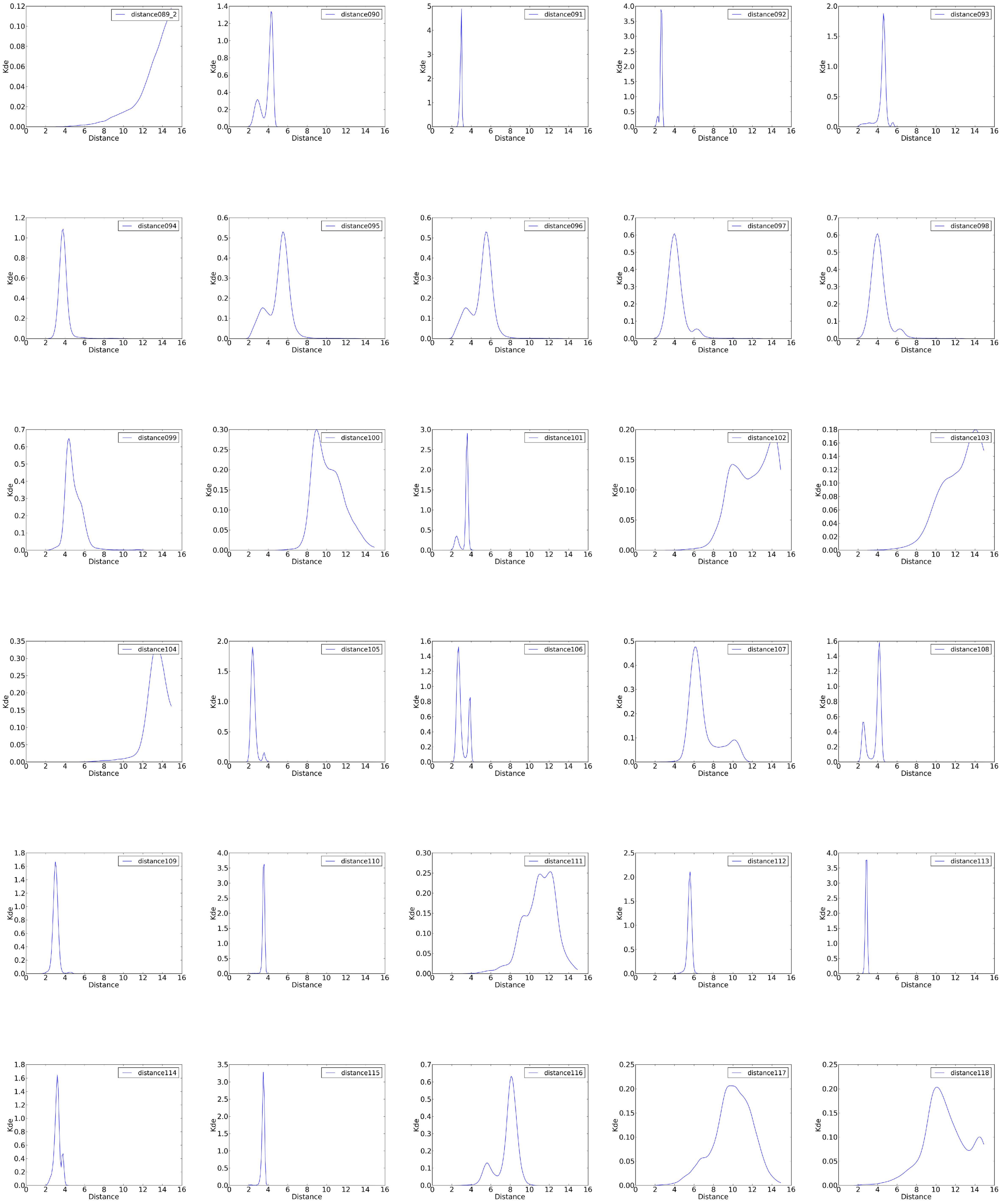
distance089_2
distance090
distance091
distance092
distance093
distance094
distance095
distance096
distance097
distance098
distance099
distance100
distance101
distance102
distance103
distance104
distance105
distance106
distance107
distance108
distance109
distance110
distance111
distance112
distance113
distance114
distance115
distance116
distance117
distance118
Kde
Distance

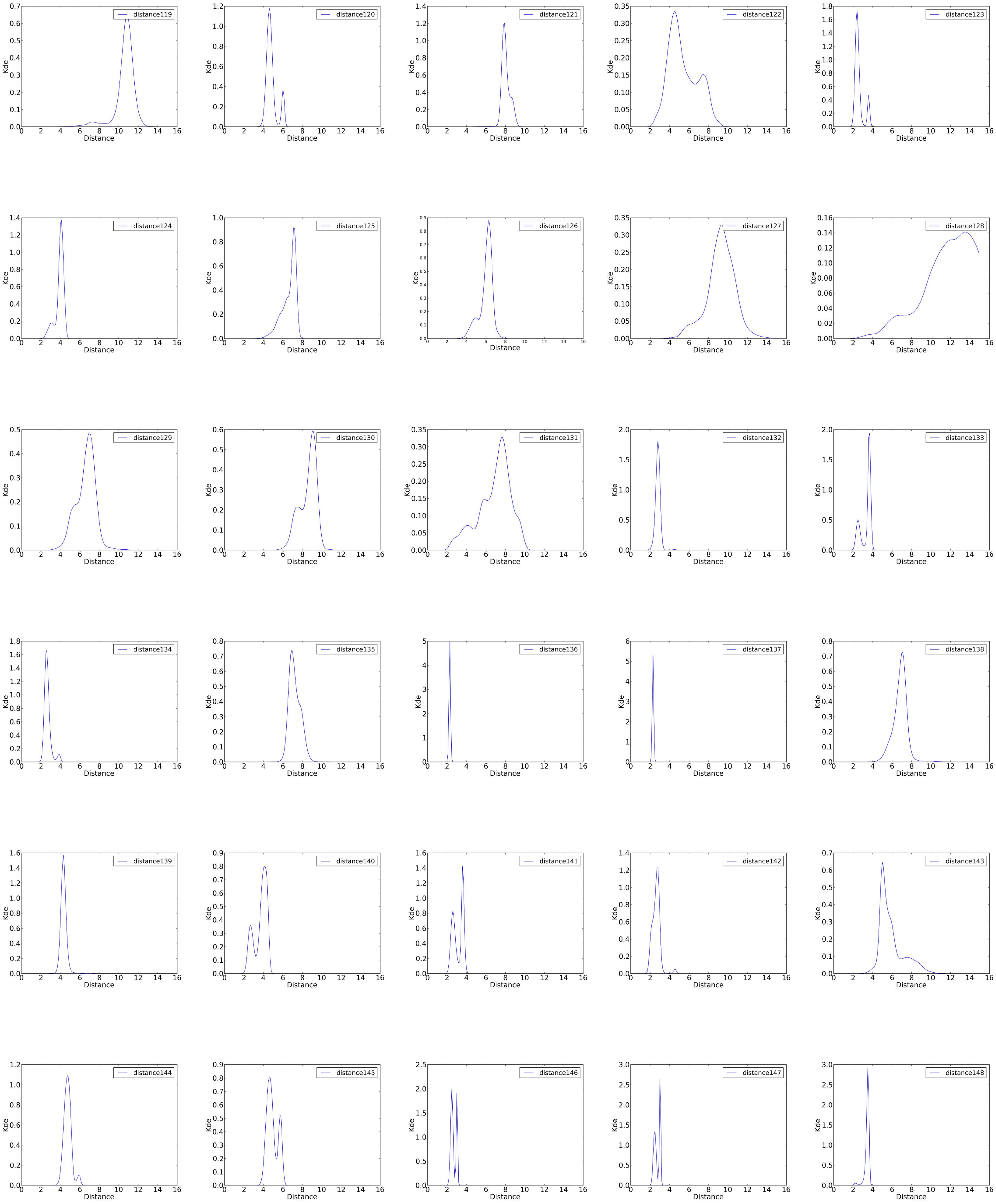
distance119
distance120
distance121
distance122
distance123
distance124
distance125
distance126
distance127
distance128
distance129
distance130
distance131
distance132
distance133
distance134
distance135
distance136
distance137
distance138
distance139
distance140
distance141
distance142
distance143
distance144
distance145
distance146
distance147
distance148
Kde
Distance

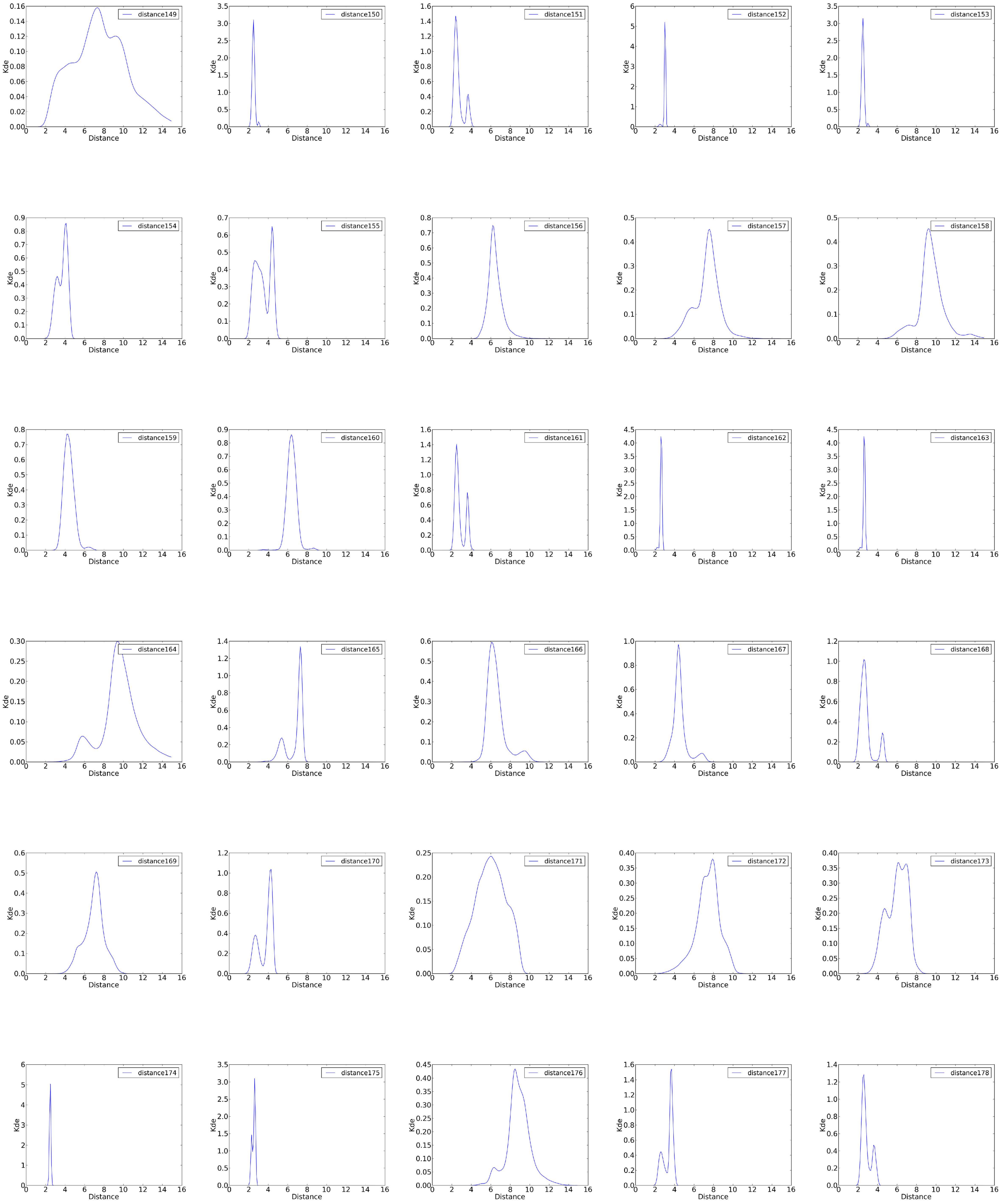
distance149
distance150
distance151
distance152
distance153
distance154
distance155
distance156
distance157
distance158
distance159
distance160
distance161
distance162
distance163
distance164
distance165
distance166
distance167
distance168
distance169
distance170
distance171
distance172
distance173
distance174
distance175
distance176
distance177
distance178
Kde
Distance

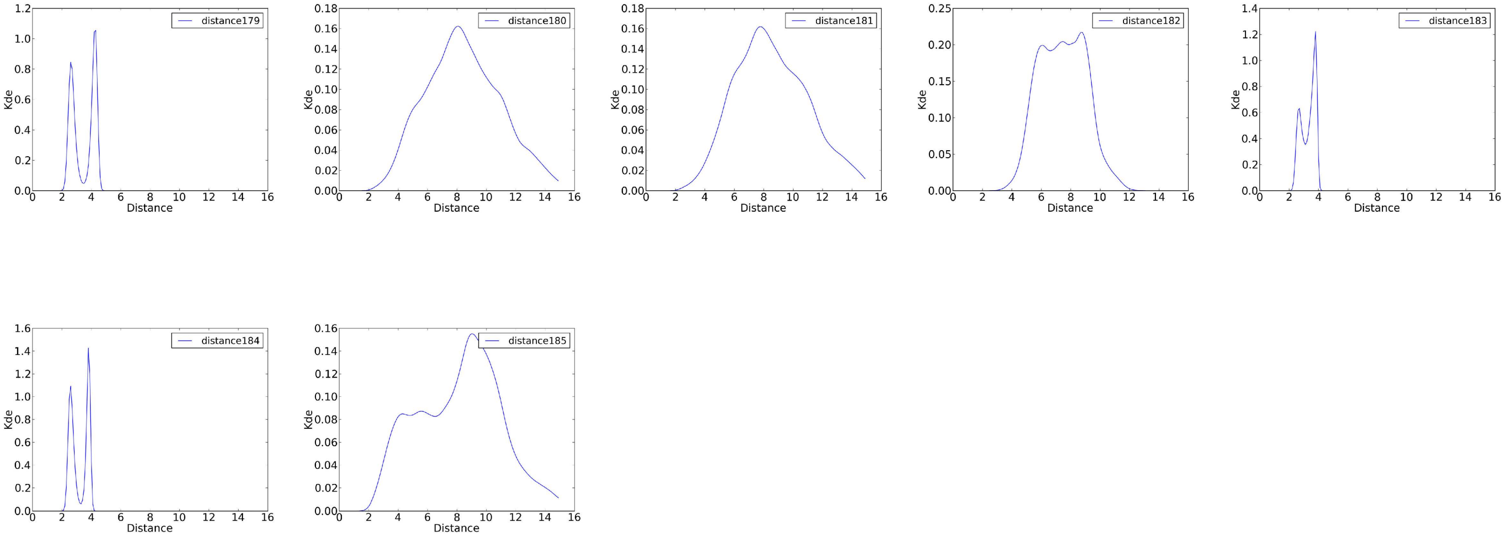


**Figure S4.** Graphical representation of the distance distribution for each of the 190 atoms pairs involved in NOE measuraments in the present work, being the peptide bond prior to Pro[9] in *trans*.

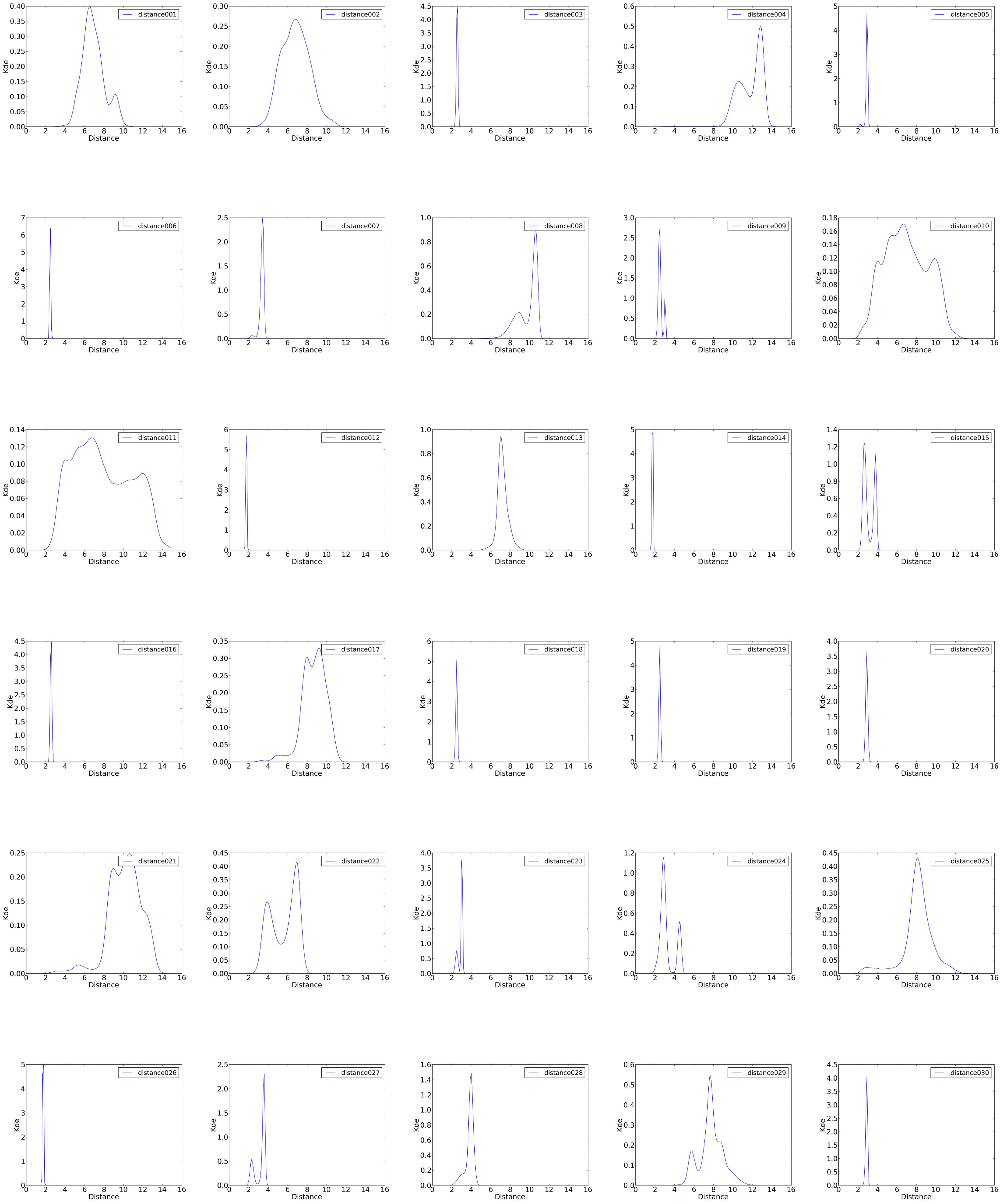
distance001
distance002
distance003
distance004
distance005
distance006
distance007
distance008
distance009
distance010
distance011
distance012
distance013
distance014
distance015
distance016
distance017
distance018
distance019
distance020
distance021
distance022
distance023
distance024
distance025
distance026
distance027
distance028
distance029
distance030
Kde
Distance

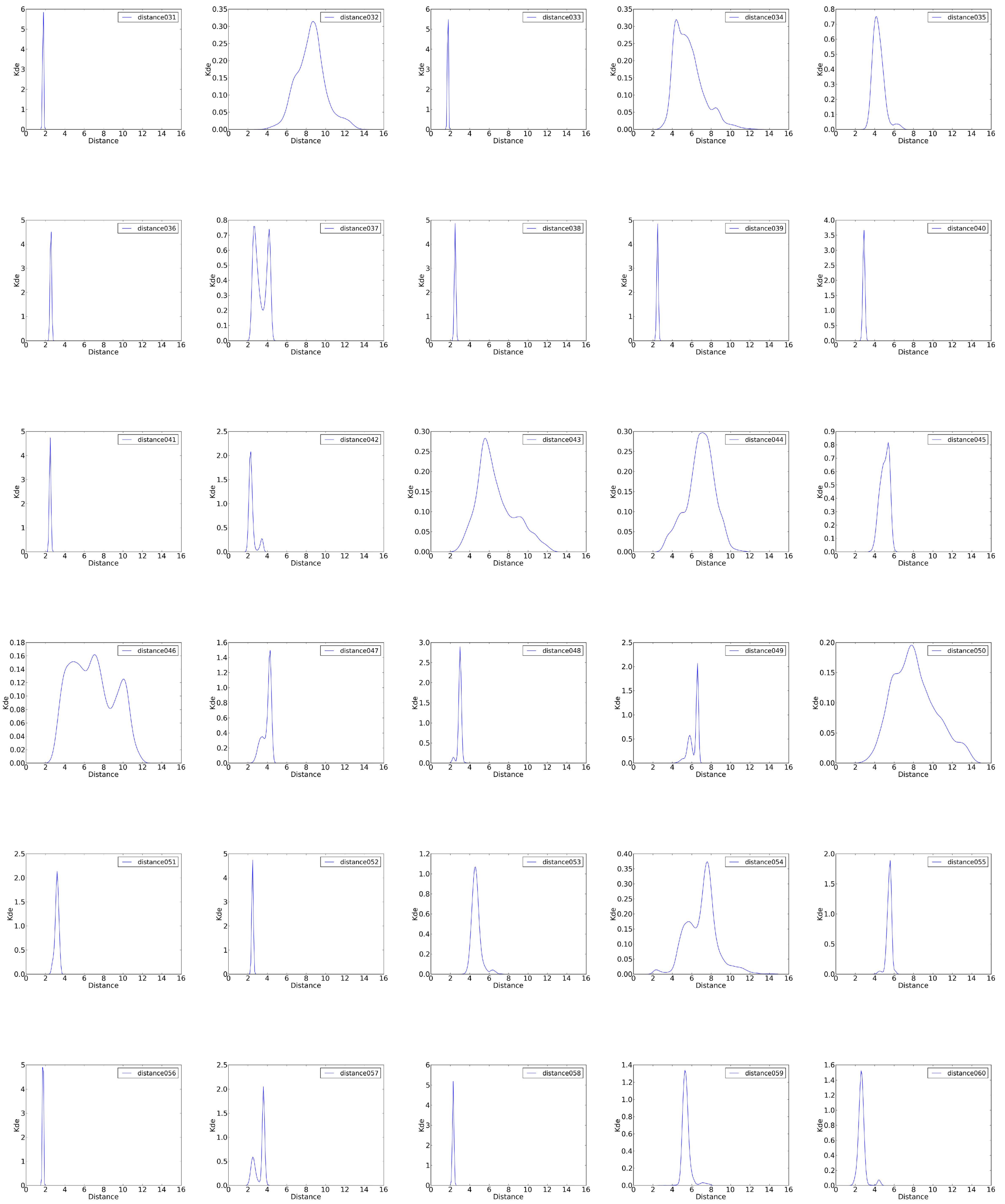
distance031
distance032
distance033
distance034
distance035
distance036
distance037
distance038
distance039
distance040
distance041
distance042
distance043
distance044
distance045
distance046
distance047
distance048
distance049
distance050
distance051
distance052
distance053
distance054
distance055
distance056
distance057
distance058
distance059
distance060
Kde
Distance

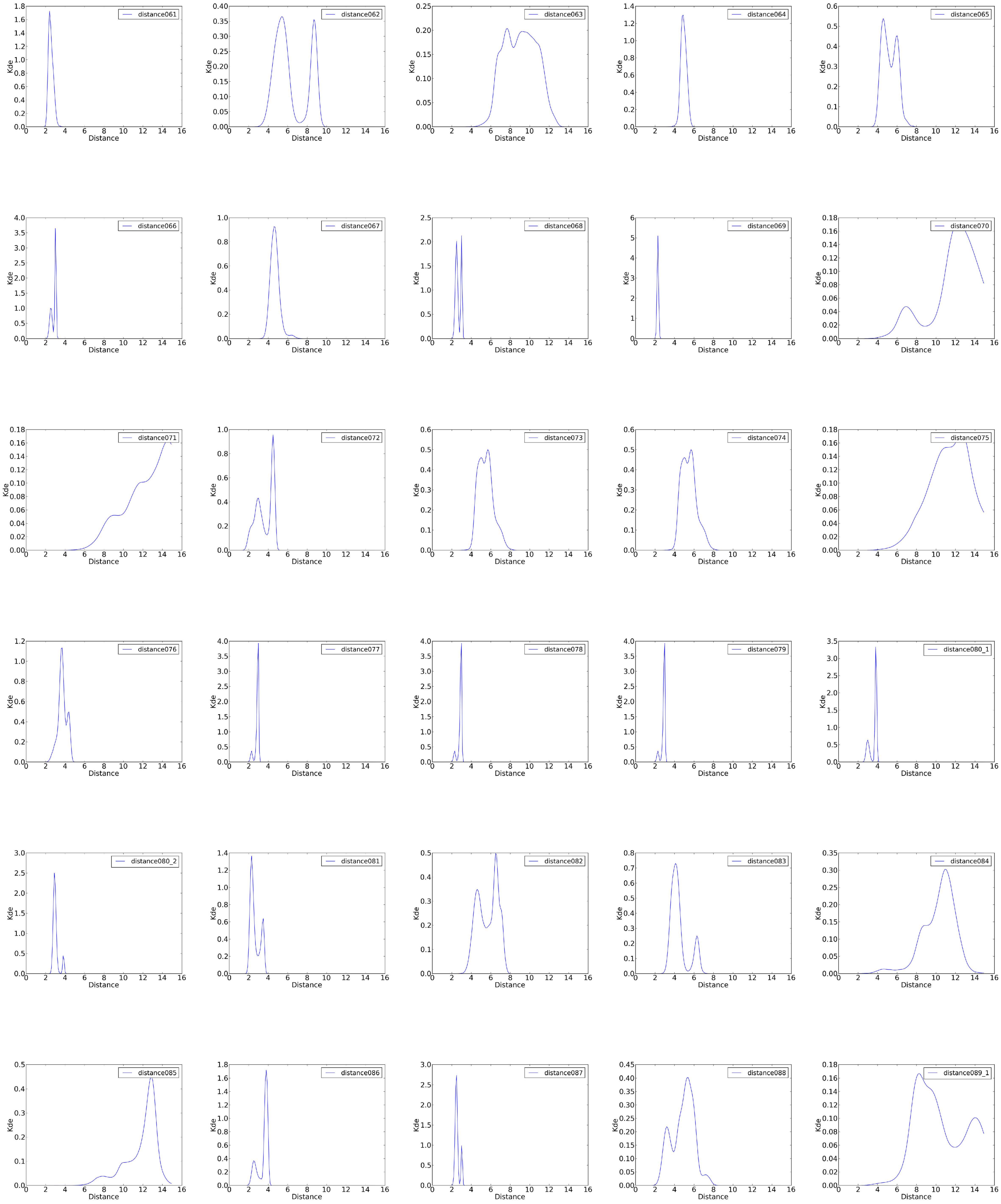
distance061
distance062
distance063
distance064
distance065
distance066
distance067
distance068
distance069
distance070
distance071
distance072
distance073
distance074
distance075
distance076
distance077
distance078
distance079
distance080_1
distance080_2
distance081
distance082
distance083
distance084
distance085
distance086
distance087
distance088
distance089_1
Kde
Distance

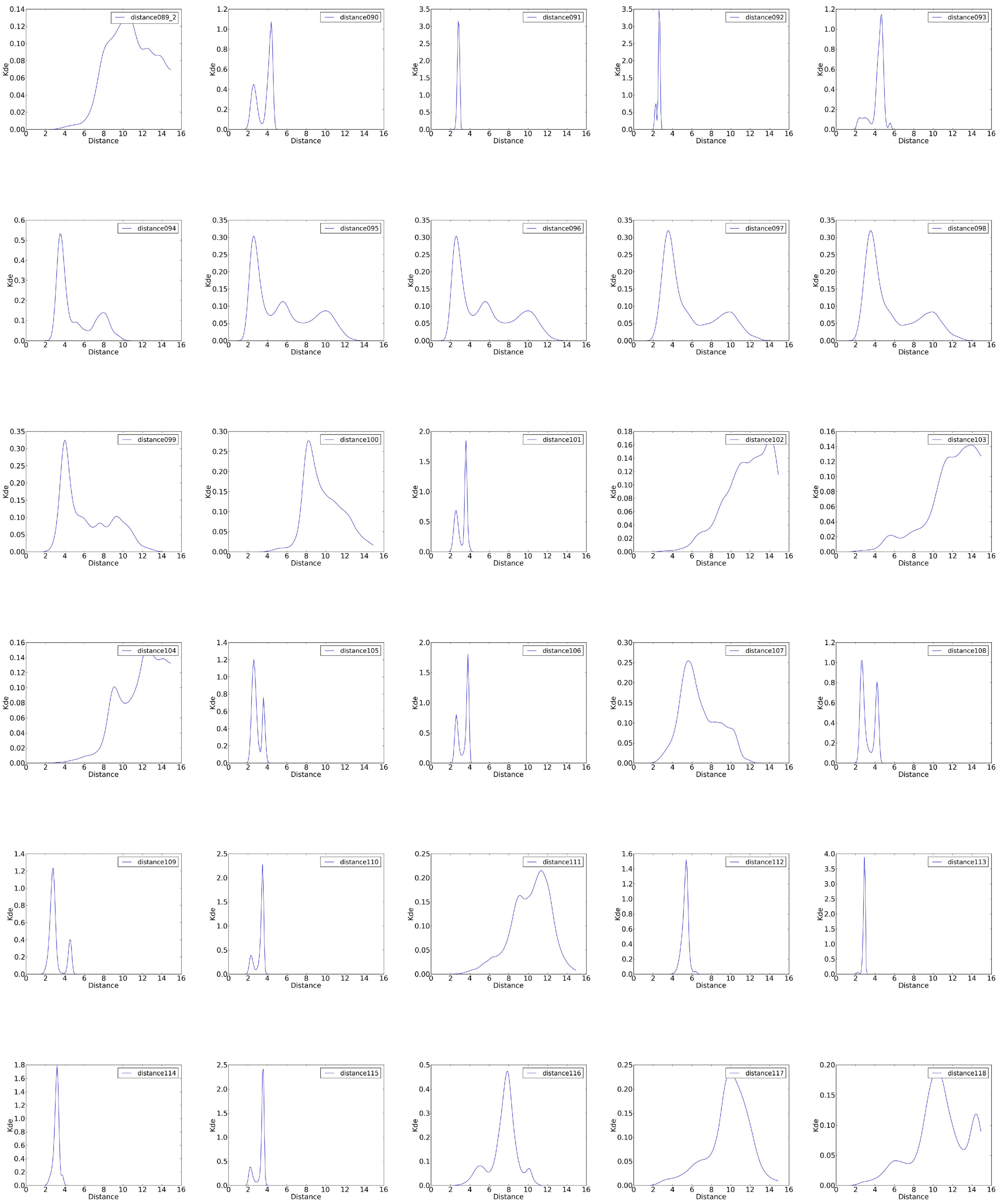

distance089_2
distance090
distance091
distance092
distance093
distance094
distance095
distance096
distance097
distance098
distance099
distance100
distance101
distance102
distance103
distance104
distance105
distance106
distance107
distance108
distance109
distance110
distance111
distance112
distance113
distance114
distance115
distance116
distance117
distance118
Kde
Distance

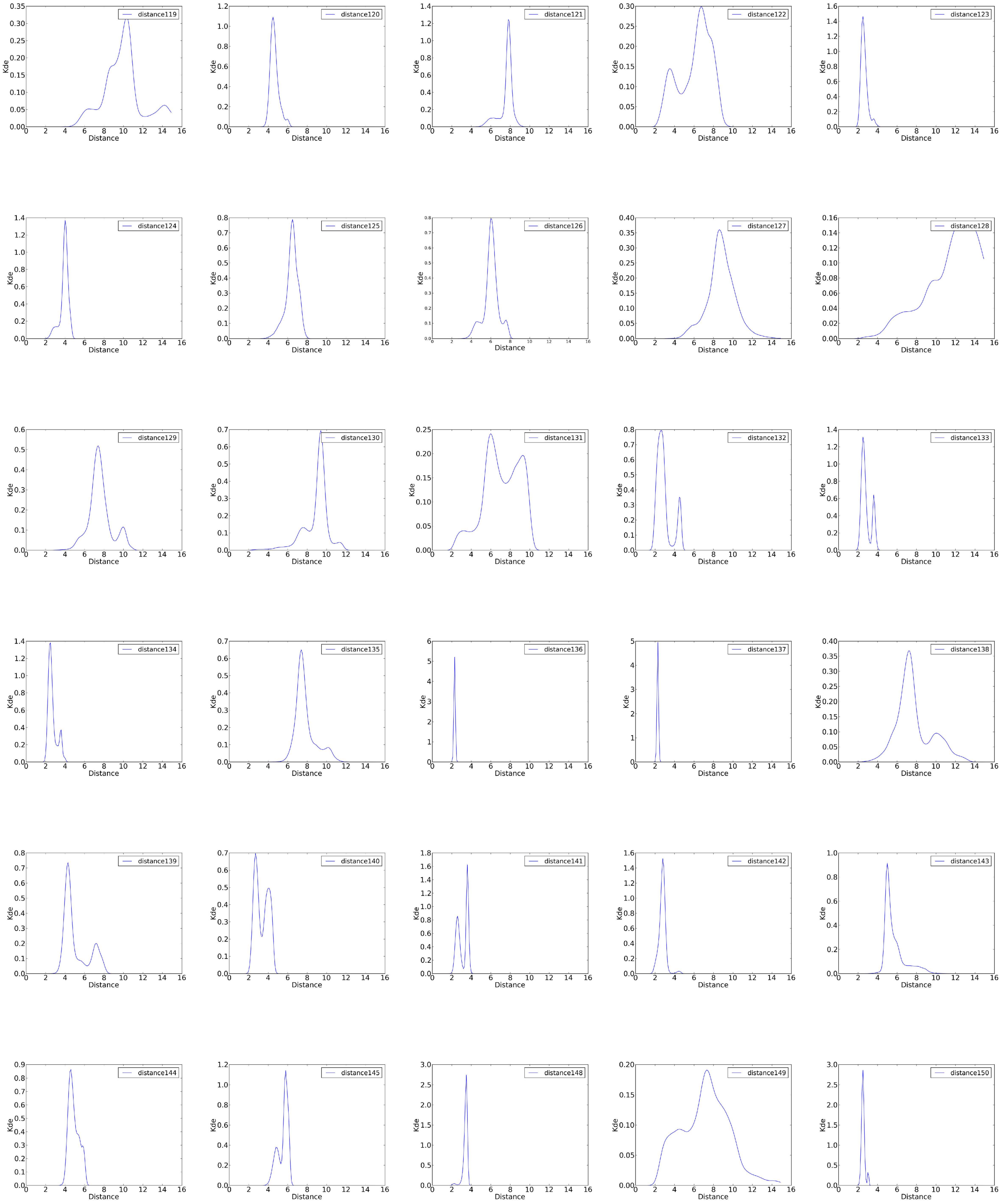
distance119
distance120
distance121
distance122
distance123
distance124
distance125
distance126
distance127
distance128
distance129
distance130
distance131
distance132
distance133
distance134
distance135
distance136
distance137
distance138
distance139
distance140
distance141
distance142
distance143
distance144
distance145
distance148
distance149
distance150
Kde
Distance

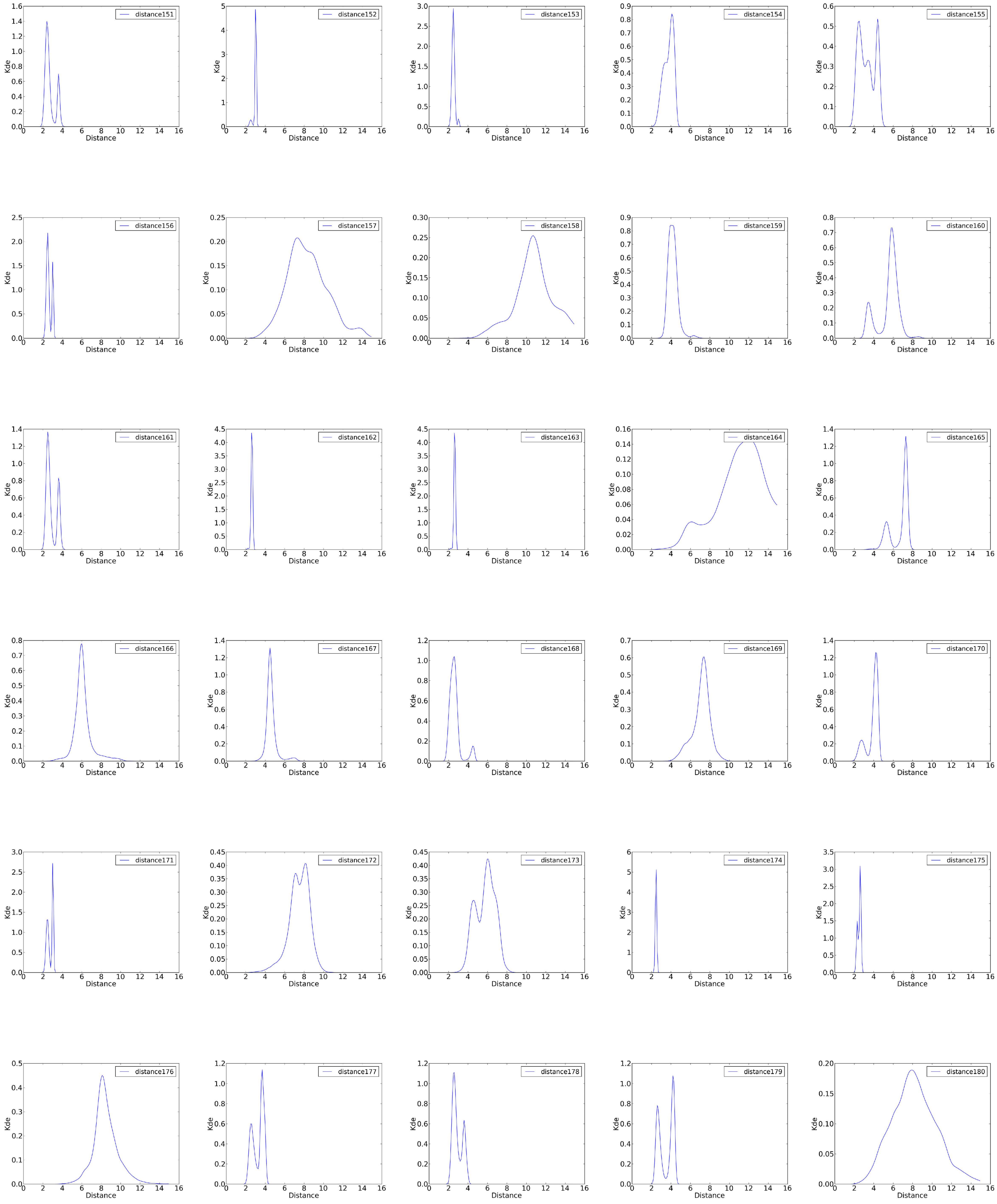
distance151
distance152
distance153
distance154
distance155
distance156
distance157
distance158
distance159
distance160
distance161
distance162
distance163
distance164
distance165
distance166
distance167
distance168
distance169
distance170
distance171
distance172
distance173
distance174
distance175
distance176
distance177
distance178
distance179
distance180
Kde
Distance

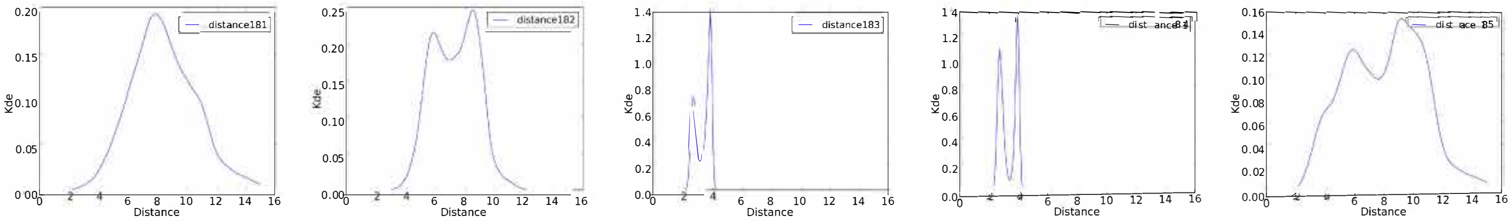


**Figure S5.** Graphical representation of the distance distribution for each of the 190 atoms pairs involved in NOE measurements in the present work, being the peptide bond prior to Pro$^{9}$ in *cis*.

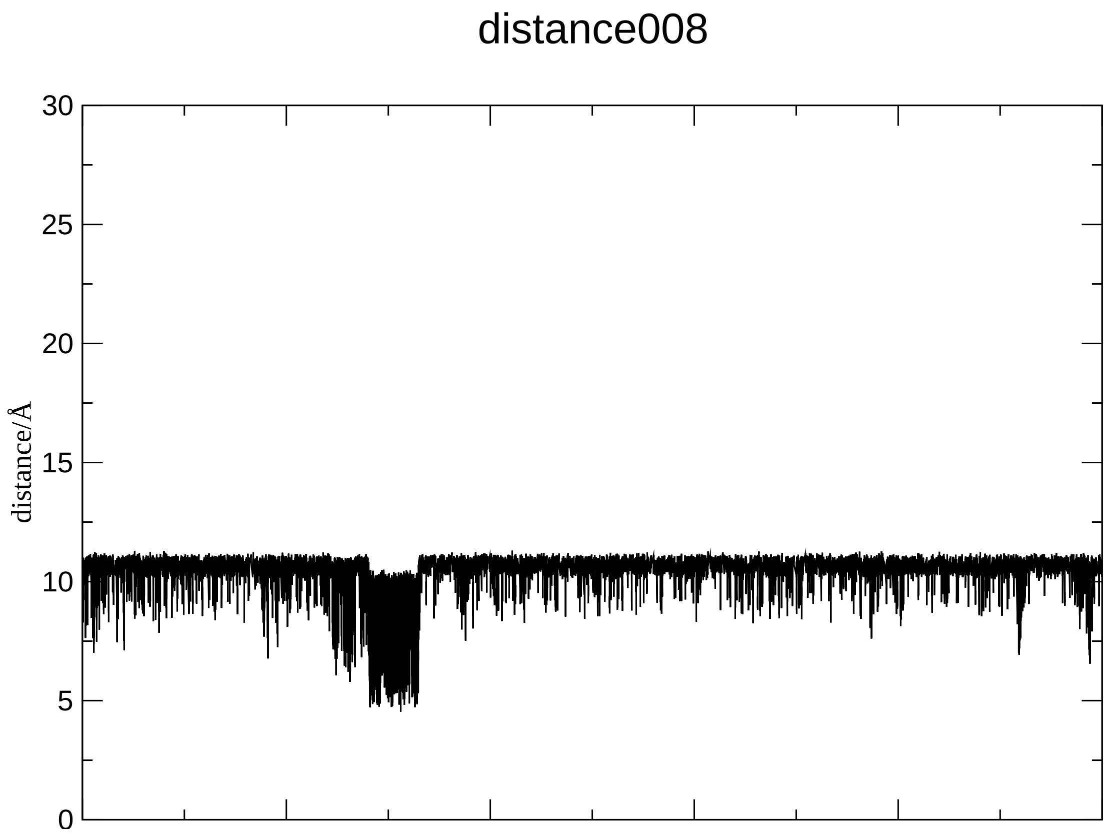

distance008
30
25
20
15
10
5
0
distance/Å


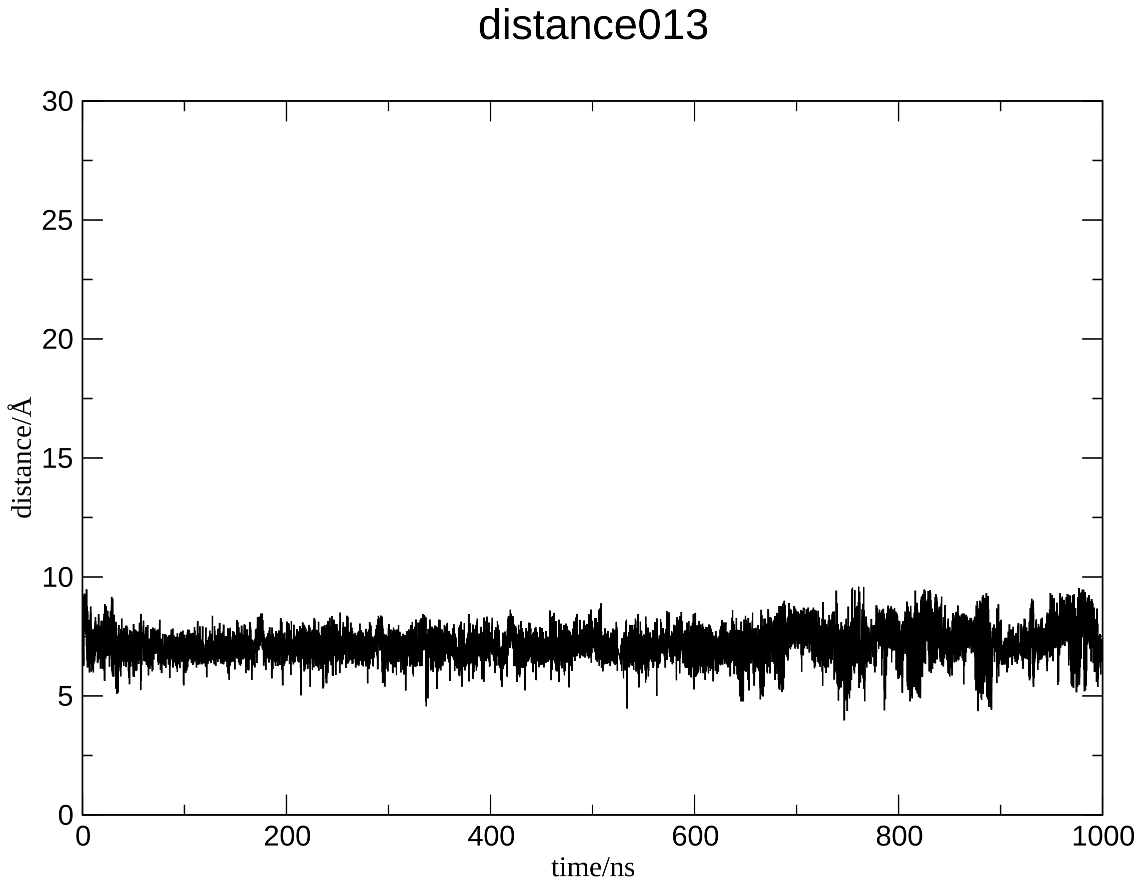

distance013
30
25
20
15
10
5
0
0
200
400
600
800
1000
distance/Å
time/ns

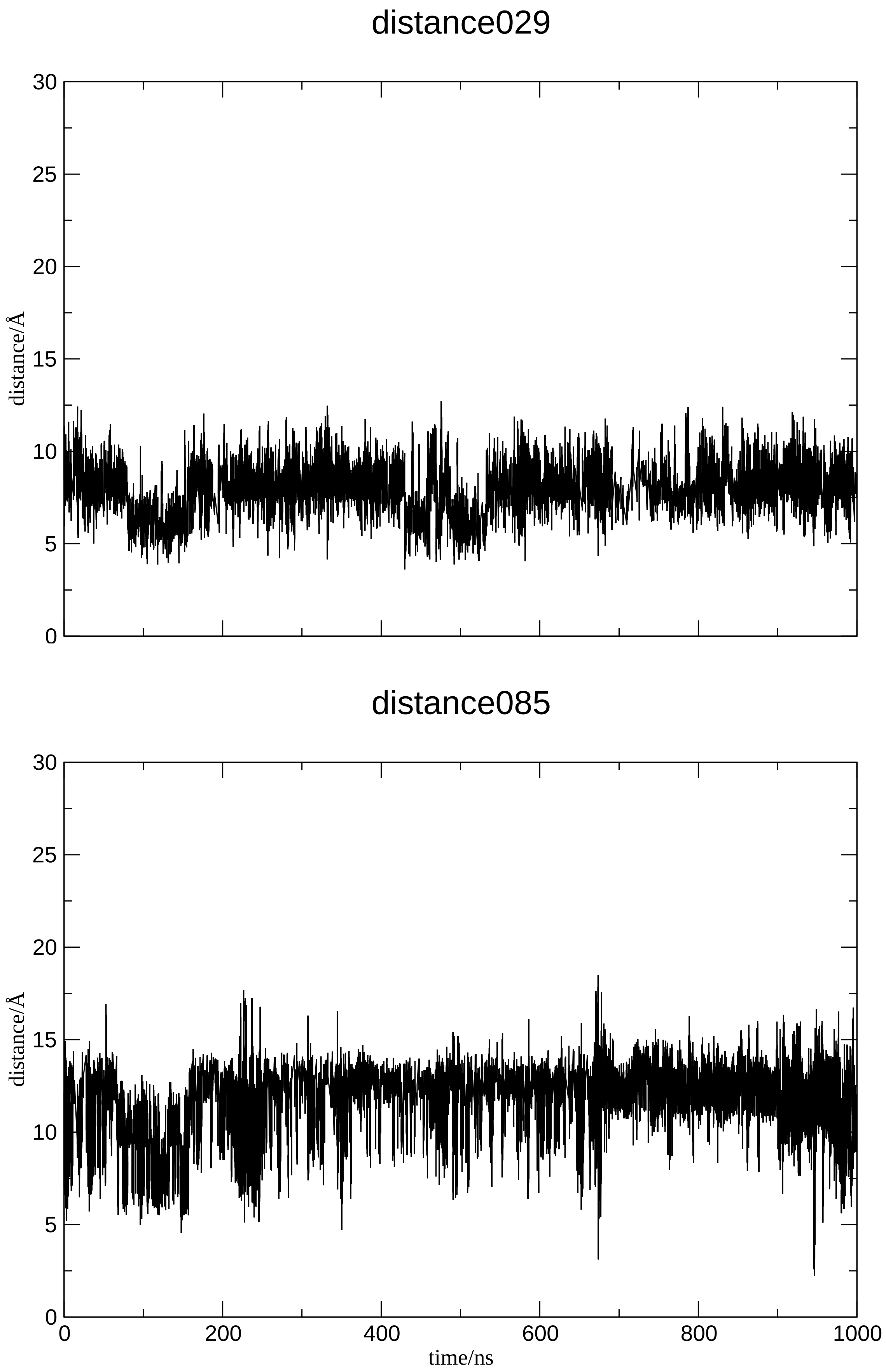
distance029
distance/Å
30
25
20
15
10
5
0
distance085
distance/Å
0
200
400
600
800
1000
time/ns

distance100

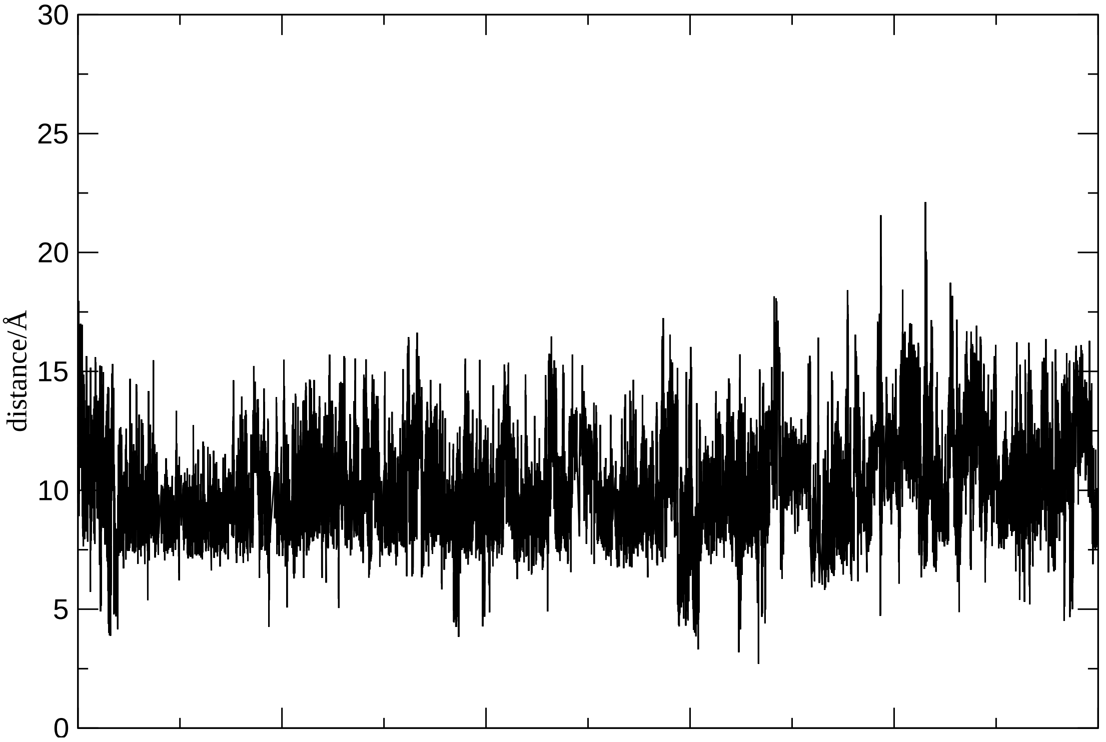


distance119

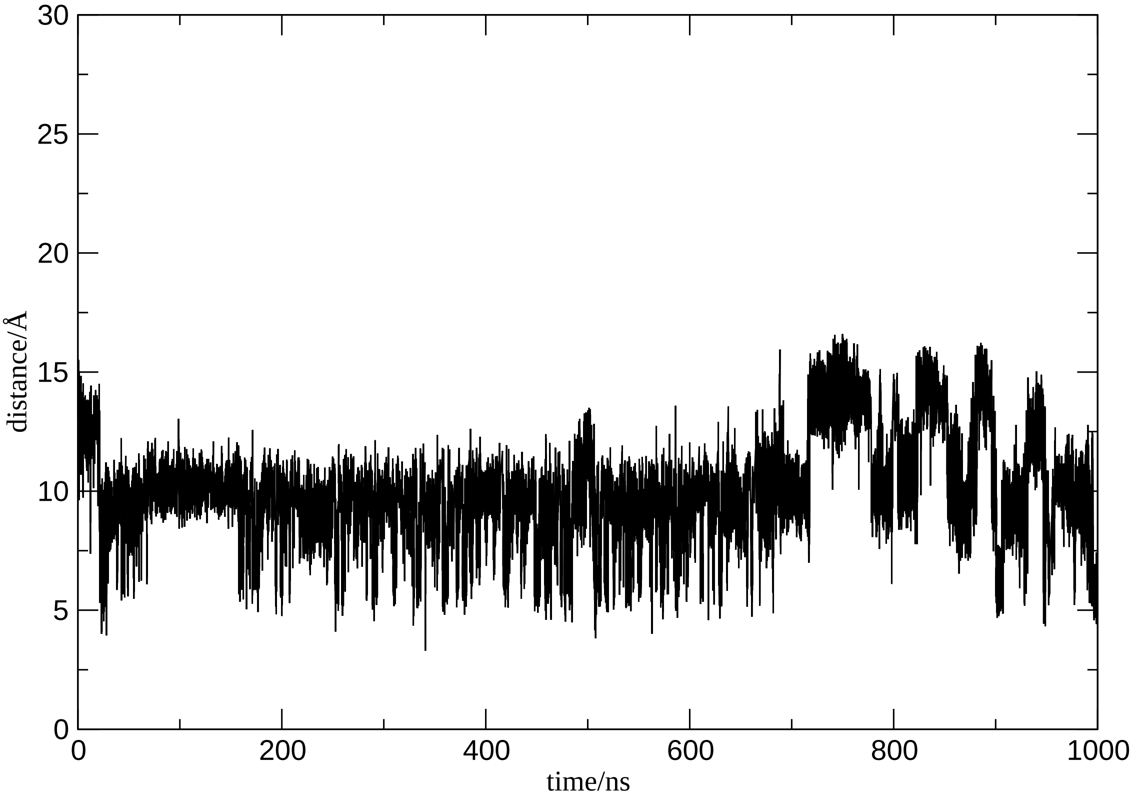

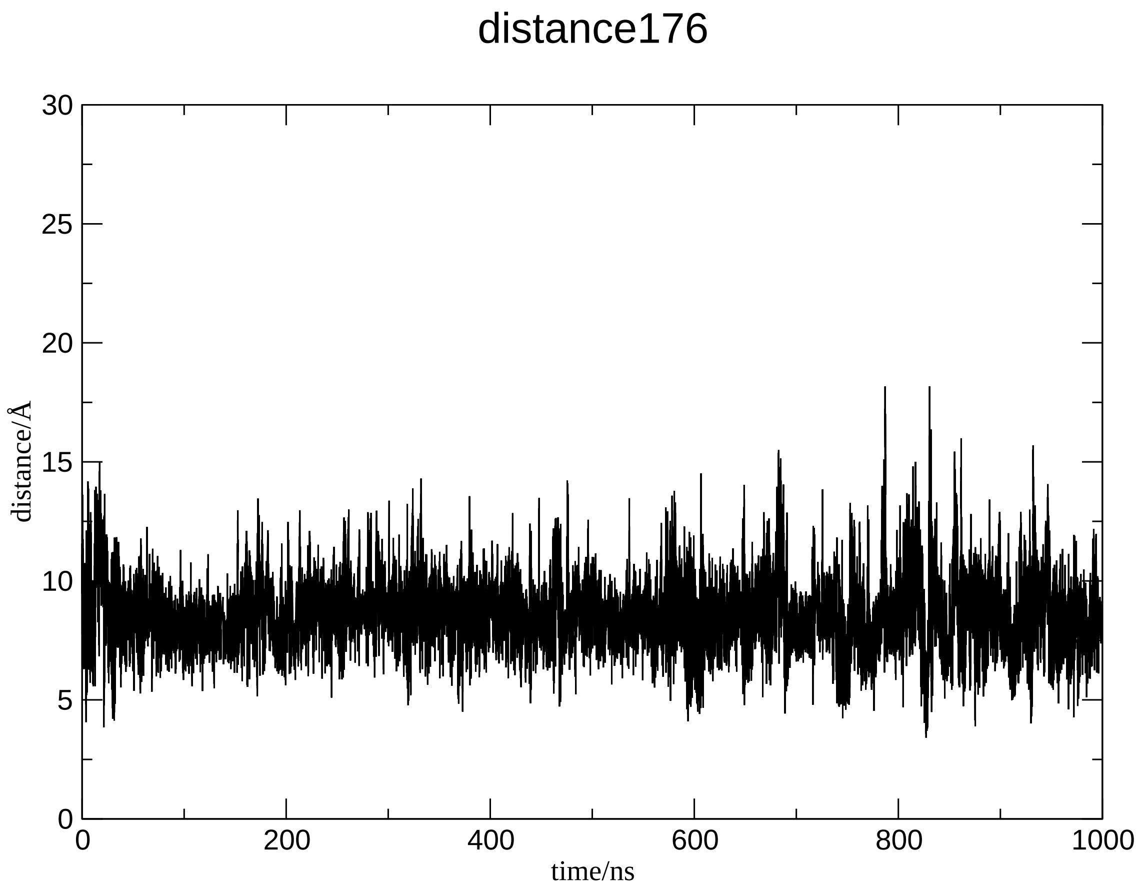


**Figure S6**. Time evolution of the distances not satisfied by any of the two structures along the MD trajectory.

A)

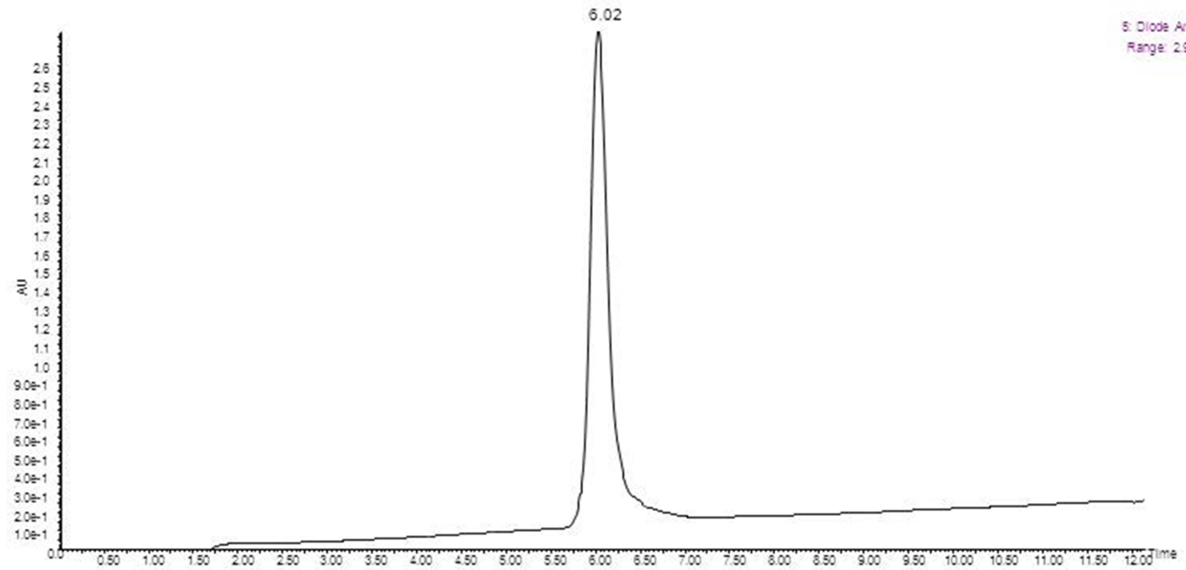


B)

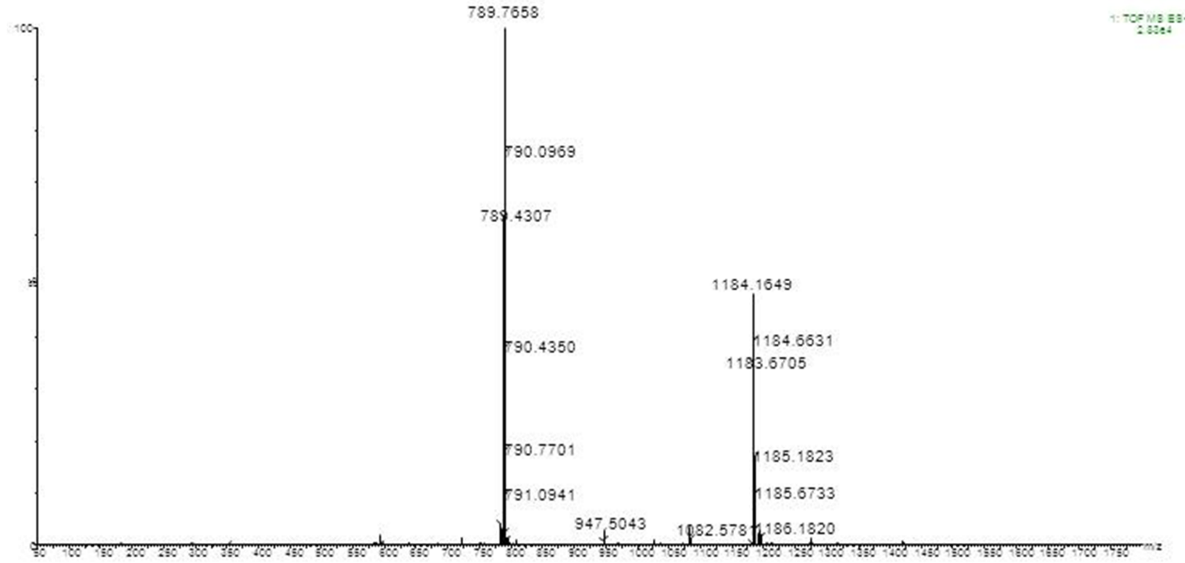


**Figure S7**. UPLC-MS characterization of RE-E1P47 peptide. (A) Peptide Elution was performed on an Acquity UPLC BEH C18 column (2.1×100 mm, 1.7 μm) with a linear gradient of 5%-100% solvent B (20mM formic acid in ACN) into solvent A (20mM formic acid in water) over 10min at 0.3mL/min. (B) The mass spectrum was recorded in positive ion mode in the m/z 50-1800 range. Calculated m/z: $[M+2H]^{+2}$=1184.15, $[M+3H]^{+3}$=789.77; experimental m/z: $[M+2H]^{+2}$= 1184.16, $[M+3H]^{+3}$= 789.77

A)

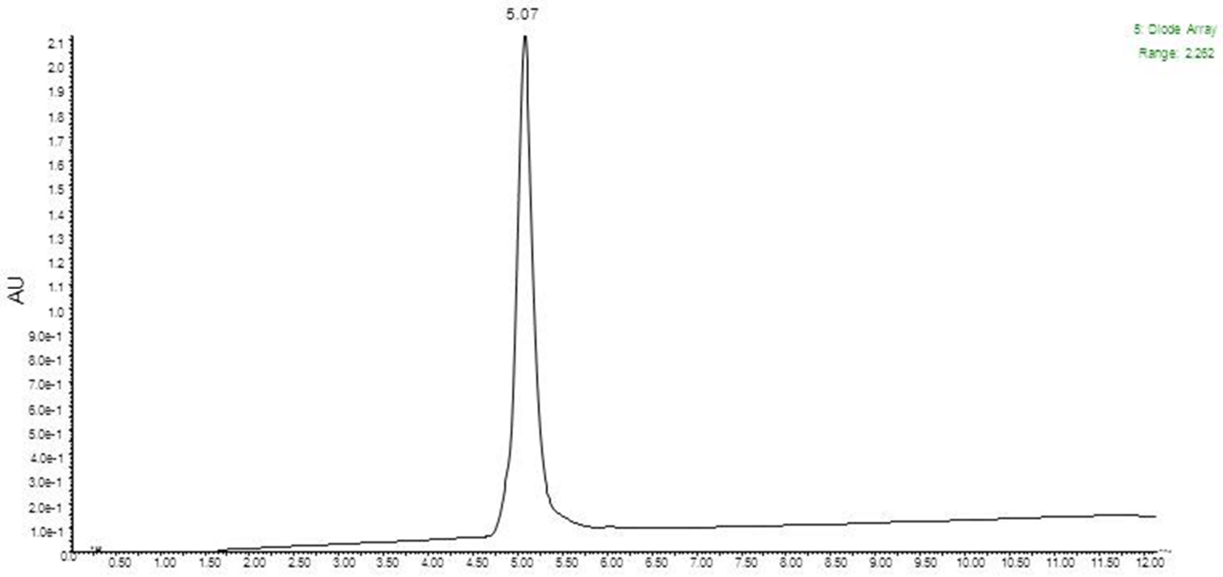


B)

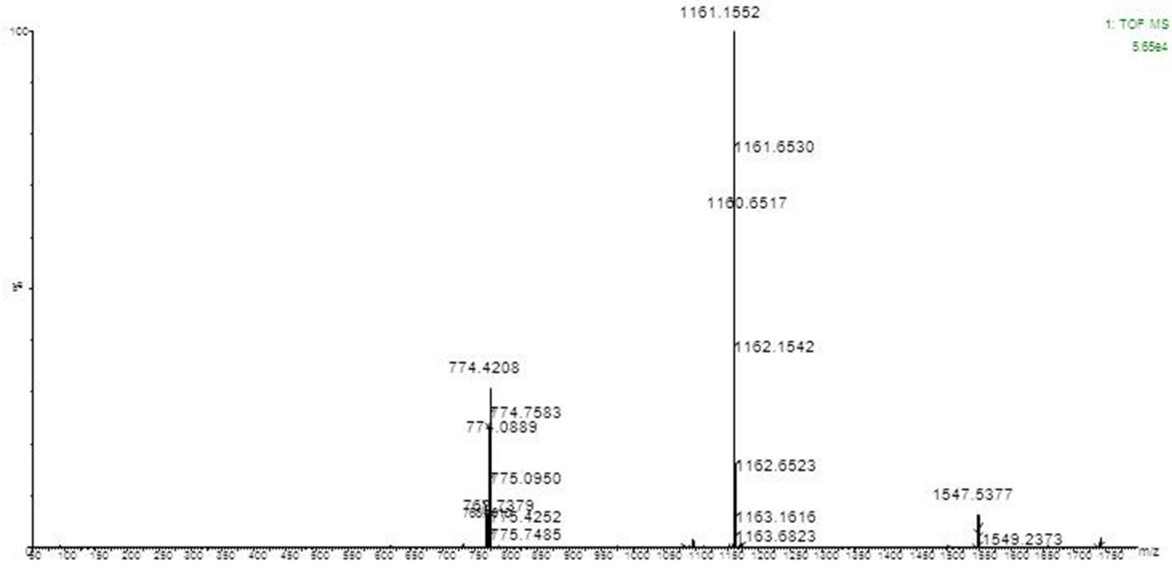


**Figure S8**. UPLC-MS characterization of StP1-E1P47 peptide. (A) Peptide Elution was performed on an Acquity UPLC BEH C18 column (2.1×100 mm, 1.7 μm) with a linear gradient of 5%-100% solvent B (20mM formic acid in ACN) into solvent A (20mM formic acid in water) over 10min at 0.3mL/min. (B) The mass spectrum was recorded in positive ion mode in the m/z 50-1800 range. Calculated m/z: $[M+2H]^{+2}$=1161.14, $[M+3H]^{+3}$=774.43; experimental m/z: $[M+2H]^{+2}$= 1161.15, $[M+3H]^{+3}$= 774.42

A)

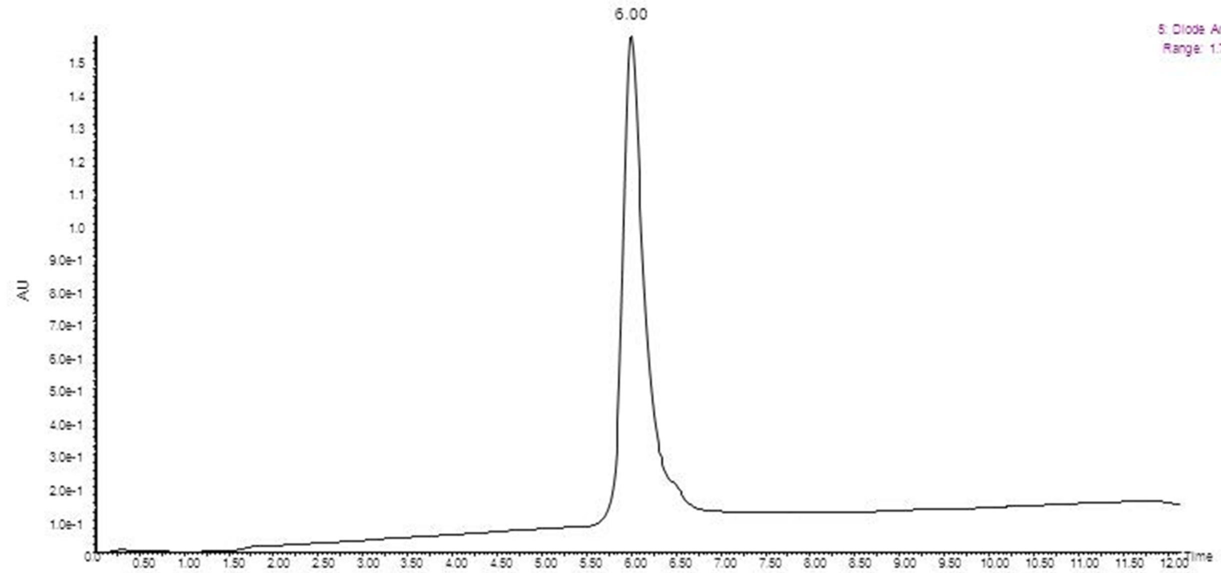


B)

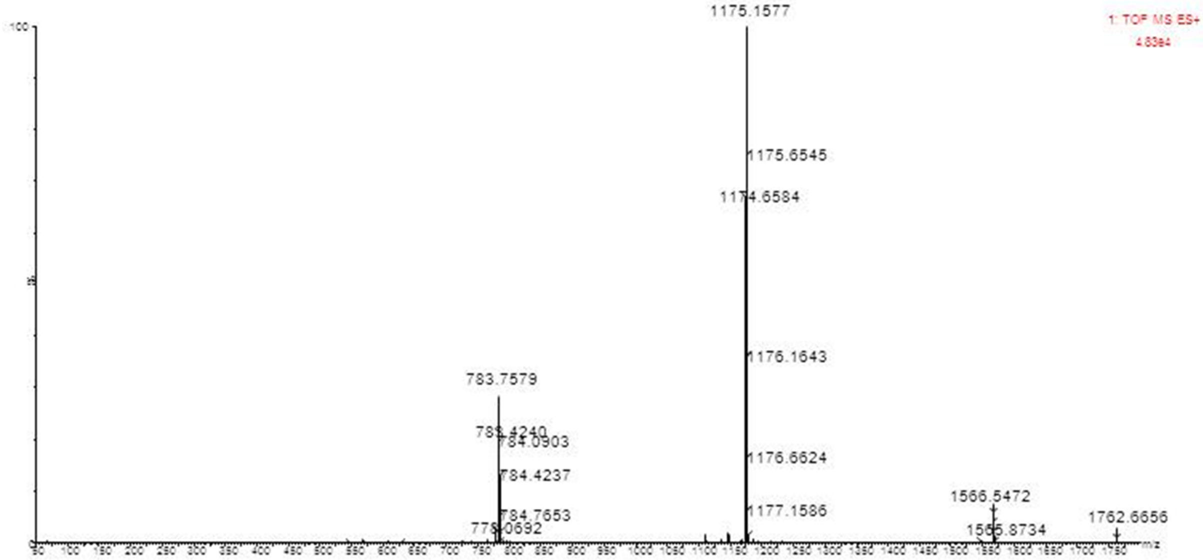


**Figure S9**. UPLC-MS characterization of StP2-E1P47 peptide. (A) Peptide Elution was performed on an Acquity UPLC BEH C18 column (2.1×100 mm, 1.7 µm) with a linear gradient of 5%-100% solvent B (20mM formic acid in ACN) into solvent A (20mM formic acid in water) over 10min at 0.3mL/min. (B) The mass spectrum was recorded in positive ion mode in the m/z 50-1800 range. Calculated m/z: $[M+2H]^{+2}$=1175.13, $[M+3H]^{+3}$=783.76; experimental m/z: $[M+2H]^{+2}$= 1175.16, $[M+3H]^{+3}$= 783.76
